# Enhancing prime editing efficiency and flexibility with tethered and split pegRNAs

**DOI:** 10.1093/procel/pwac014

**Published:** 2022-07-15

**Authors:** Ying Feng, Siyuan Liu, Qiqin Mo, Pengpeng Liu, Xiao Xiao, Hanhui Ma

**Affiliations:** School of Biotechnology, East China University of Science and Technology, Shanghai 200237, China; Gene Editing Center, School of Life Science and Technology, ShanghaiTech University, Shanghai 200135, China; Gene Editing Center, School of Life Science and Technology, ShanghaiTech University, Shanghai 200135, China; Gene Editing Center, School of Life Science and Technology, ShanghaiTech University, Shanghai 200135, China; Department of Molecular, Cell and Cancer Biology, University of Massachusetts Medical School, Worcester, MA 01655, USA; School of Biotechnology, East China University of Science and Technology, Shanghai 200237, China; Gene Editing Center, School of Life Science and Technology, ShanghaiTech University, Shanghai 200135, China

**Dear Editor**,

Most human genetic diseases arise from mutations such as insertion, deletion, or point mutations (Landrum et al., 2016). CRISPR-Cas system has been repurposed to correct pathogenic mutations in a variety of genetic diseases (Choi et al., 2022). There are many concerns about using CRISPR-mediated double-stranded DNA breaks (DSBs) for therapeutic purposes, primarily due to off-targeted mutations (Kosicki et al., 2018). Nevertheless, base editing cannot correct deletions, insertions, or some point mutations such as transversion mutations. Prime editing has its advantages of precisely correct point mutations, small insertions, or deletions in animal cells (Anzalone et al., 2019) and plants (Lin et al., 2020). However, prime editing efficiency varies among genomic sites or cell types (Chen et al., 2021; Nelson et al., 2022). The reasons for cause variable efficiency of the prime editing are yet to be identified. Prime editing requires the assembly of the PE (Cas9 nickase fused to reverse transcriptase) and pegRNA to be PE-pegRNA complex. PE-pegRNA complex searches and nicks target DNA at the non-template strand, followed by reverse transcription, and mutagenesis is done by 3ʹ-flap resolution (Anzalone et al., 2019). Thus, it is crucial to optimize pegRNA and PE-pegRNA complex for higher PE efficiency and precision.

Robust prime editing is required to satisfy a series of conditions, such as stable and properly folded pegRNAs, effective assembly of PE-pegRNA complex, targeting to genomic loci, efficient reverse transcription, and correct editing. Unstructured RNA sequence appended to the 3ʹ-end of sgRNA destabilizes the sgRNAs (Nelson et al., 2022). The PEs consist of a *Streptococcus pyogenes* Cas9 nickase-H840A with C-terminal fusion of an MMLV (PE2), and a pegRNA which includes a prime binding site (PBS) and a reverse transcription template (RTT) at 3ʹ-terminal of sgRNA. PBS and RTT at the 3ʹ-terminal of pegRNA are easy to be partially degraded, resulting in truncated pegRNAs. The truncated pegRNAs can still search and recognize the target sites, but not be able to complete the correct editing due to loss of the PBS or RTT-PBS (Nelson et al., 2022). In addition, pegRNA circularization might also result in self-inhibition and compromise the PE efficiency (Liu et al., 2021). We have shown that the dynamics of CRISPR DNA targeting limits genome editing efficiency (Ma et al., 2016).

Here we used CRISPR-based genome imaging (Ma et al., 2016, 2018) to compare the target efficiency of CRISPR-based GE (Genome Editor) and PE (Prime Editor). Fluorescent Cas9-sgRNA complex effectively targeted to chromosome 3-specific tandem repeats (C3) allows to be visualized under microscopy in U2OS cells (Fig. S1) (Ma et al., 2016). As shown in Fig. S1B and S1C, 2–4 bright foci were observed in the GE system but not the PE system suggesting that 3ʹ-terminal RTT-PBS of pegRNA resulted in low target efficiency of PE. We added the stem-loop aptamer MS2 (Convery et al., 1998) at the 3ʹ-terminal of pegRNAs (pegRNA-MS2) and found that visualization of C3 loci was recovered (Fig. S1B and (S1C). We assume that the 3ʹ-terminal pegRNA tethered to Cas9 nickase will stabilize the PE-pegRNA complex. We fused tandem MS2 coat protein (tdMCP) to the N-terminal of Cas9 for binding 3ʹ-terminal MS2 at the engineered pegRNA. As we can see in Fig. S1B and S1C, C3 labeling was maintained. The low targeting efficiency of canonical PE suggests that inefficient targeting of genomic loci may compromise the PE efficiency. On the contrary, the recovery of C3 loci visualization in 3ʹ-terminal MS2 tagged pegRNA or tethered to Cas9 nickase indicates the engineered pegRNA or tethered to Cas9 nickase may improve the PE efficiency.

To distinguish from the canonical prime editor (PE), we named the PE system with 3ʹ-stem-loop MS2, PP7, Csy4, and BoxB tagged pegRNA to be stem-loop PE (sPE), and the system with pegRNA-MS2, PP7, Csy4, and BoxB tethered to Cas9 nickase-MMLV was named tethered PE (tPE) (Fig. 1A). First, we tested sPE-MS2, PP7, Csy4, and BoxB and tPE-MS2, PP7, Csy4, and BoxB (Urbanek et al., 2014) on the PE efficiency using PE3 in HEK293FT cells at *RUNX1* (+5 G·C to T·A). Use of either sPEs or tPEs improved correct editing efficiency with no significant change in edit/indel ratios (Figs. 1B, S4A and S4D). We also compared ePE-Mpknot, ePE-EvopreQ1 (Nelson et al., 2022) on the same loci in HEK293FT cells. The correct editing efficiency of sPE-MS2, PP7, Csy4, BoxB, and tPE-MS2, Csy4 on this loci are higher than ePE-Mpknot, EvopreQ1 (Fig. 1B). We also tested the small insertion and deletion efficiency by tPE-MS2, PP7, Csy4, and BoxB at *RUNX1*_with +1 ATG insertion (Fig. 1C) or_+1 CGA deletion (Fig. 1D) resulting in a 4.9- or 2.7-fold increase on average in PE efficiency with no significant change in edit/indel ratios overall (Fig. S4B, S4C, S4E, and S4F) relative to that of canonical PE in HEK293FT cells. Therefore, we chose MS2 appended at the 3ʹ-terminal of pegRNA on the PE efficiency using PE3 in HEK293FT cell at 10 loci including *SRD5A3* (+2 C·G to A·T), *DYRK1A* (+1 C·G to G·C), *HDAC1* (+1 C·G to G·C), *BCL11A* (+1 C·G to A·T), *GFAP* (+1 A·T to T·A), *RUNX1* (+5 G·C to T·A), *JAK2* (+1 C·G to T·A), *SRD5A1* (+1 C·G to A·T), *DMD* (+1 T·A to C·G), and *EED* (+1 A·T to T·A) (Fig. 1E and 1F). Use of either sPE-MS2 or tPE-MS2 resulted in a 1.8- or 1.9-fold average improvement in PE efficiency relative to that of canonical PE across tested sites in HEK293FT cells (Fig. 1G) with no significant change in edit/indel ratios overall (Fig. S3A and S3B). We also test the insertion efficiency of *DNMT1*_+4 A, +4 AC, and +4 ACT and deletion efficiency of *DNMT1*_+4 G, +4 GG, and +4 GGG using tPE-MS2. The PE efficiency by tPE-MS2 increased 2–3 folds on average without significant change in edit/indel ratios (Fig. S5).

**Figure 1.**
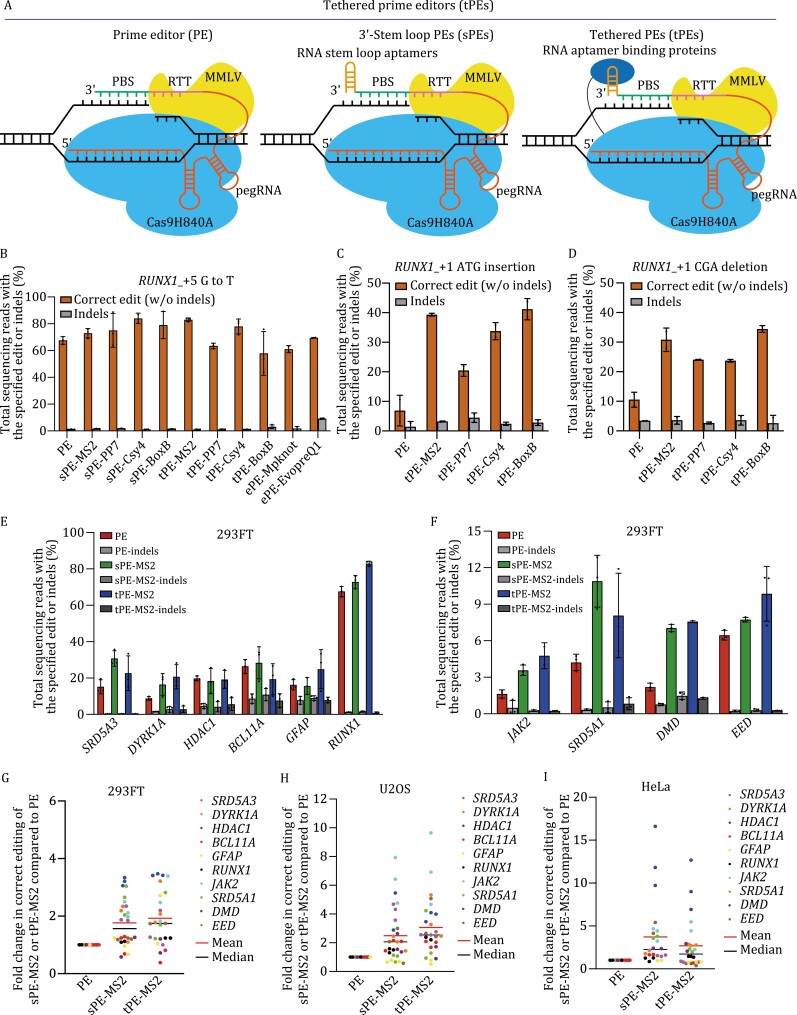
pegRNA with 3ʹ-RNA aptamers or tethered to Cas9 nickase enhance targeting and editing efficiency. (A) The prime editing (PE) complex consists of a Streptococcus pyogenes Cas9 nickase-H840A with C-terminal fusion of an MMLV, and a pegRNA which includes a prime binding site (PBS) and a reverse transcription template (RTT) at 3ʹ-terminal of sgRNA. 3ʹ-stem-loop PE (sPE)-MS2 was generated by appending an MS2 stem-loop aptamer to the 3ʹ-terminal of pegRNA. The tethered PE (tPE)-MS2 was generated by fusing tandem MS2 coat protein (tdMCP) to the N-terminal of Cas9 for cognate RNA aptamers in sPEs. (B) Comparison of editing efficiency between PE, sPE-MS2, PP7, Csy4, BoxB, tPE-MS2, PP7, Csy4, BoxB, ePE-Mpknot, EvopreQ1 mediated point mutation of *RUNX1*_+5 G·C to T·A using PE3 in HEK293FT cells. (C) Editing efficiency for *RUNX1*_+1 ATG insertion of tPE-MS2, PP7, Csy4, and BoxB. (D) Editing efficiency for *RUNX1*_+1 CGA deletion of tPE-MS2, PP7, Csy4, and BoxB, compared to canonical PE (dashed line). (E) The efficiency of PE, sPE-MS2, and tPE-MS2 mediated point mutation of *SRD5A3_*+2 C·G to A·T, *DYRK1A_*+1 C·G to G·C, *HDAC1_*+1 C·G to G·C, *BCL11A*_+1C·G to A·T, *GFAP*_+1A·T to T·A, and *RUNX1*_+5 G·C to T·A using PE3 in HEK293FT cells. (F) The efficiency of PE, sPE-MS2, and tPE-MS2 mediated point mutation *JAK2_*+1 C·G to T·A, *SRD5A1*_+1 C·G to A·T, *DMD_*+1 T·A to C·G, and *EED_*+1 A·T to T·A using PE3 in HEK293FT cells. Comparison of editing efficiencies of canonical PE, sPE-MS2, or tPE-MS2 for point mutation at 10 loci in HEK293FT cells (G), U2OS (H), and HeLa cells (I). Values were calculated from the data presented in Figs. 1 and S2. Dots indicate the average of three biological replicates and bars indicate the grand median. Data and error bars in (B–F) indicate the mean and standard deviation of three independent biological replicates.

PE efficiency varies in different cell types (Chen et al., 2021; Nelson et al., 2022). To ensure that the improvement in PE efficiency by sPEs or tPEs was not limited to HEK293FT cells, we tested the above 10 loci with sPE-MS2 or tPE-MS2 in U2OS (Fig. S2A and S2B) and HeLa cells using PE3 (Fig. S2C and S2D). In either U2OS or HeLa, sPE-MS2 or tPE-MS2 resulted in improvements in editing efficiency compared to canonical PE, averaging 2.5- or 3.1-fold higher editing in U2OS cells (Fig. 1H) and 3.7- or 2.7-fold higher editing in HeLa cells (Fig. 1I), with no significant change in edit/indel ratios overall (Fig. S3A and S3D). These results indicate that sPE and tPE can enhance PE efficiency in different cell types. We examined off-target editing by sPE-MS2 and tPE-MS2 for *DYRK1A*, *RUNX1*, *BCL11A*, *SRD5A3*, and *JAK2* loci in HEK293FT cells. The off-target sites were predicted by Cas-OFFinder (Bae et al., 2014). Average <0.1% off-target prime editing was detected in canonical PE, sPE-MS2, and tPE-MS2 at the predicted off-target sites for each protospacer of *DYRK1A*, *RUNX1*, *BCL11A*, *SRD5A3*, or *JAK2* in HEK293FT cells (Fig. S6).

To make the PE more flexible, we split the modified pegRNA in the tPEs into sgRNA and prime RNA (pRNA), and generate split pegRNA prime editors (SnPEs) (Fig. 2A). To stabilize pRNA, we generate circular prime RNA (cpRNA) by Tornado circRNA expression system (Litke and Jaffrey, 2019) (Fig. S7). Briefly, we split pegRNA-MS2, PP7, BoxB, and Csy4 into sgRNA and pRNA, resulting in pRNA-5ʹ-MS2, pRNA-3ʹ-MS2, pRNA-c(ircular)-MS2, pRNA-5ʹ-PP7, pRNA-3ʹ-PP7, pRNA-c-PP7, pRNA-5ʹ-BoxB, pRNA-5ʹ-Csy4 (Fig. 2A). Very low SnPE activity was observed when using control pRNA without MS2 or PP7 in U2OS, HEK293FT, and HeLa cells (Fig. 2B–E). The PE efficiency is comparable to canonical PE when SnPE-5ʹ-MS2, SnPE-c-MS2, SnPE-5ʹ-PP7 were used. It shows 82.3% of canonical PE activity for SnPE-5ʹ-MS2, 72.1% for SnPE-c-MS2 and 59.4% for SnPE-5ʹ-PP7 and 46.0% for SnPE-c-PP7 in HEK293FT cells (Fig. 2B–E). The efficiency became much lower when SnPE-3ʹ-MS2 (31.7%) or SnPE-3ʹ-PP7 (18.3%) was used (Fig. 2B–E). The highest PE efficiency was found when using SnPE-5ʹ-PP7 (79.5% of canonical PE) in U2OS and SnPE-5ʹ-MS2 (59.8%) in HeLa cells. The edit/indel ratios of SnPEs are slightly lower than canonical PE in all three cell types, which is in concord with the level changes of PE activities (Fig. S8A–F).

**Figure 2.**
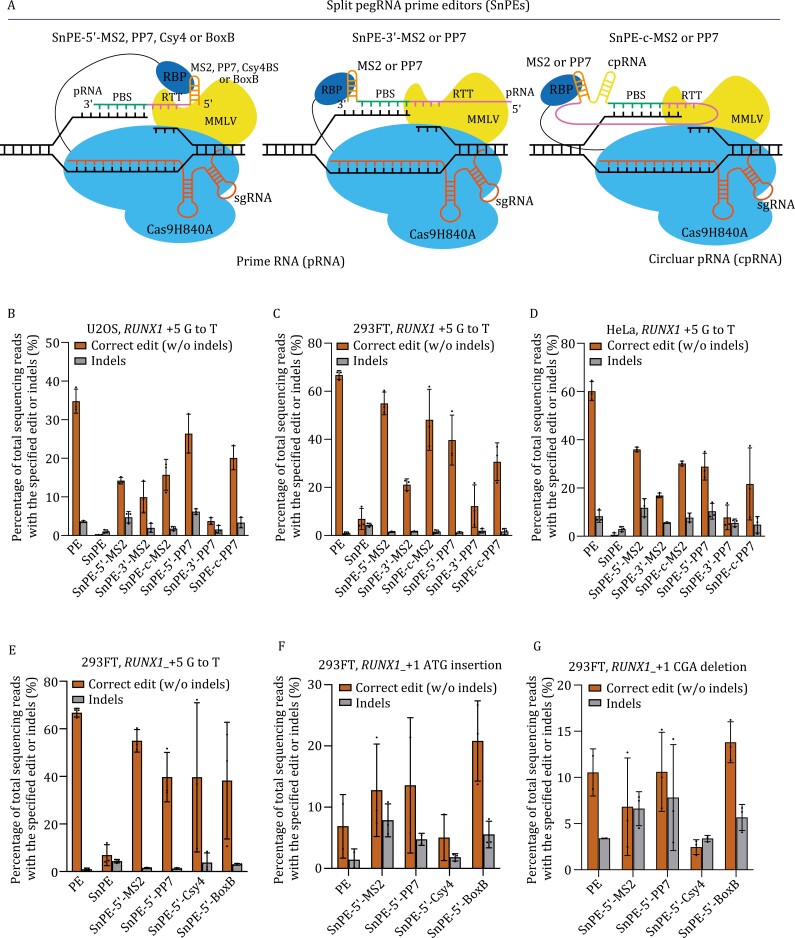
Split pegRNA prime editing maintains PE activity by tethering prime RNA to Cas9. (A) Schematics of split pegRNA prime editors. The split pegRNA prime editor (SnPE) consists of a Cas9 nickase-H840A fused with C-terminal MMLV and N-terminal RNA binding proteins (RBPs), a sgRNA, and a separated prime RNA (pRNA). pRNA consists of a prime binding site (PBS), a reverse transcription template (RTT) and a RNA stem-loop aptamer such as MS2 or PP7. SnPE-5ʹ or 3ʹ-MS2 or PP7 consists of RBP-Cas9 nickase-MMLV, a pRNA bearing MS2 or PP7 and a sgRNA. RBP at the N-terminal Cas9 binds the RNA aptamer MS2 or PP7. SnPE-c-MS2 or PP7 includes the circular pRNA (cpRNA) bearing MS2 or PP7 generated by the tornado circular RNA expression system. Efficiency of SnPE-5ʹ-MS2 or PP7, SnPE-3ʹ-MS2 or PP7, SnPE-c-MS2, or PP7 were tested at RUNX1_+5 G·C to T·A using PE3 in U2OS cells (B), HEK293FT cells (C), and HeLa cells (D). (E) Efficiency of PE, SnPE, SnPE-5ʹ-Com, SnPE-5ʹ-BoxB, and SnPE-5ʹ-Csy4 at *RUNX1*_+5 G·C to T·A using PE3 in HEK293FT cells. (F) Efficiency of SnPE-5ʹ-MS2 or PP7, SnPE-5ʹ-MS2 or PP7, SnPE-5ʹ-Csy4 and SnPE-5ʹ-BoxB were tested at *RUNX1*_+1 ATG insertion using PE3 HEK293FT cells. (G) Efficiency of SnPE-5ʹ-MS2 or PP7, SnPE-5ʹ-MS2 or PP7, SnPE-5ʹ-Csy4, and SnPE-5ʹ-BoxB were tested at *RUNX1*_+1 CGA deletion using PE3 HEK293FT cells. Data and error bars in (B–G) indicate the mean and standard deviation of three independent biological replicates.

We further tested whether pRNA-5ʹ-BoxB or pRNA-5ʹ-Csy4 could also improve the *RUNX1*_+1 ATG insertion efficiency and *RUNX1*_+1 CGA deletion efficiency. The SnPE-5ʹ-MS2, PP7, BoxB showed higher activities for *RUNX1*_+1 ATG insertion than canonical PE in HEK293FT cells, particularly the SnPE-5ʹ-BoxB showed 2-fold increase in the efficiency for *RUNX1*_+1 ATG insertion (Fig. 2F). SnPE-5ʹ-PP7, BoxB also showed higher activities for *RUNX1*_+1 CGA deletion than canonical PE (Fig. 2G) in HEK293FT cells. The edit/indel ratios changes are in concord with the level changes of PE activities (Fig. S8G–L). These results indicate SnPEs maintain the activity and increase the flexibility of prime editing.

The prime editing has the advantages for point mutations, small deletions, and insertions. However, the instability or misfolding of pegRNAs may have limited its applications for direct insertion of bigger size fragment such as >100 nucleotides. It will be interesting to test whether sPEs or tPEs will allow for the installation of DNA fragments with hundreds of nucleotides. There are several dual pegRNA strategies to increase the efficiency and precision of small or large deletions, small fragment insertions (Anzalone et al., 2022; Choi et al., 2022). Large fragment insertion has also been achieved by the combination of dual-pegRNA mediated small insertions and recombinase-mediated site-specific genomic integration (Anzalone et al., 2022). It will be intriguing to test whether sPEs or tPEs will benefit these dual-pegRNA systems since sPEs and tPEs showed better targeting efficiency than canonical PEs.

Tethered PEs offer the opportunity to liberate the RTT-PBS unit from the pegRNAs and spatiotemporally control the PEs. pegRNA in tPEs was separated to be conventional sgRNA and prime RNA containing PBS, RTT, and RNA aptamer resulting in SnPEs. One of the potential applications of SnPEs is more readily prepared by chemical synthesis of split pegRNAs due to the smaller sizes of sgRNA and pRNA. Synthetically modified sgRNA and prime RNAs may further enhance the PE efficiency. The SnPEs could also combine with inhibitors of DNA mismatch repair (MMR) (Chen et al., 2021) to further increase prime editing efficiency and precision. Separated prime RNA could be also introduced under the control of chemicals or lights (Stanton et al., 2018) and evolve the PE system to be tunable in space and time.

## Supplementary Material

pwac014_suppl_Supplementary_Material

## Footnotes

293FT cell was a gift from W. Qi Lab. We thank P.W. Zhang and L.S. Zhang for help with cell sorting and H.Z. Liu for imaging. We are grateful to Biomedical Big Data Platform L.C. Jiang and Z.Y. Song for deep sequencing. Fluorescence activated cell sorting (FACS) and DeltaVision Ultra microscopy were provided by Shanghai Institute for Advanced Immunochemical Studies (SIAIS) at Shanghaitech University. The authors have filed patent applications on sPEs, tPEs, and SnPEs through ShanghaiTech University.

This work was funded by National Natural Science Foundation of China (No. 31970591 to H. Ma), the Shanghai Pujiang program (19PJ1408000 to H. Ma) and Shanghai Science and Technology Innovation Action Plan (21JC1404800 to H. Ma).

The authors have filed patent applications on sPEs, tPEs and SnPEs through ShanghaiTech University.

The next-generation sequencing data have been deposited in the NCBI Sequence Read Archive database under the BioProject accession code PRJNA785066. All other relevant data are available from corresponding authors upon reasonable request.

Demultiplexing and base calling were both performed using bcl2fastq Conversion Software v2.18 (Illumina, Inc.). Alignment of sequencing reads to the Amplicon sequence (Table S5) was performed using CRISPResso2 (Clement et al., 2019).

## References

[CIT0001] Anzalone AV , Gao XD, Podracky CJ et al. Programmable deletion, replacement, integration and inversion of large DNA sequences with twin prime editing. Nat Biotechnol 2022;40:731–740. 34887556 10.1038/s41587-021-01133-w PMC9117393

[CIT0002] Anzalone AV , Randolph PB, Davis JR et al. Search-and-replace genome editing without double-strand breaks or donor DNA. Nature 2019;576:149–157. 31634902 10.1038/s41586-019-1711-4 PMC6907074

[CIT0003] Bae S , Park J, Kim JS. Cas-OFFinder: a fast and versatile algorithm that searches for potential off-target sites of Cas9 RNA-guided endonucleases. Bioinformatics 2014;30:1473–1475. 24463181 10.1093/bioinformatics/btu048 PMC4016707

[CIT0004] Chen PJ , Hussmann JA, Yan J et al. Enhanced prime editing systems by manipulating cellular determinants of editing outcomes. Cell 2021;184:5635–5652.e29. 34653350 10.1016/j.cell.2021.09.018 PMC8584034

[CIT0005] Choi J , Chen W, Suiter CC et al. Precise genomic deletions using paired prime editing. Nat Biotechnol 2022;40:218–226. 34650269 10.1038/s41587-021-01025-z PMC8847327

[CIT0006] Convery MA , Rowsell S, Stonehouse NJ et al. Crystal structure of an RNA aptamer-protein complex at 2.8 A resolution. Nat Struct Biol 1998;5:133–139. 9461079 10.1038/nsb0298-133

[CIT0007] Kosicki M , Tomberg K, Bradley A. Repair of double-strand breaks induced by CRISPR-Cas9 leads to large deletions and complex rearrangements. Nat Biotechnol 2018;36:765–771. 30010673 10.1038/nbt.4192 PMC6390938

[CIT0008] Landrum MJ , Lee JM, Benson M et al. ClinVar: public archive of interpretations of clinically relevant variants. Nucleic Acids Res 2016;44:D862–D868. 26582918 10.1093/nar/gkv1222 PMC4702865

[CIT0009] Lin Q , Zong Y, Xue C et al. Prime genome editing in rice and wheat. Nat Biotechnol 2020;38:582–585. 32393904 10.1038/s41587-020-0455-x

[CIT0010] Litke JL , Jaffrey SR. Highly efficient expression of circular RNA aptamers in cells using autocatalytic transcripts. Nat Biotechnol 2019;37:667–675. 30962542 10.1038/s41587-019-0090-6 PMC6554452

[CIT0011] Liu Y , Yang G, Huang S et al. Enhancing prime editing by Csy4-mediated processing of pegRNA. Cell Res 2021;31:1134–1136. 34103663 10.1038/s41422-021-00520-x PMC8486859

[CIT0012] Ma H , Tu LC, Naseri A et al. CRISPR-Cas9 nuclear dynamics and target recognition in living cells. J Cell Biol 2016;214:529–537. 27551060 10.1083/jcb.201604115 PMC5004447

[CIT0013] Ma H , Tu LC, Naseri A et al. CRISPR-Sirius: RNA scaffolds for signal amplification in genome imaging. Nat Methods 2018;15:928–931. 30377374 10.1038/s41592-018-0174-0 PMC6252086

[CIT0014] Nelson JW , Randolph PB, Shen SP et al. Engineered pegRNAs improve prime editing efficiency. Nat Biotechnol 2022;40:402–410. 34608327 10.1038/s41587-021-01039-7 PMC8930418

[CIT0015] Stanton BZ , Chory EJ, Crabtree GR. Chemically induced proximity in biology and medicine. Science 2018;359:6380. 10.1126/science.aao5902 PMC6417506 29590011

[CIT0016] Urbanek MO , Galka-Marciniak P, Olejniczak M et al. RNA imaging in living cells—methods and applications. RNA Biol 2014;11:1083–1095. 25483044 10.4161/rna.35506 PMC4615301

