# Correction to: Enhancing prime editing efficiency and flexibility with tethered and split pegRNAs

**DOI:** 10.1093/procel/pwad025

**Published:** 2023-05-25

This is a correction to: Ying Feng, Siyuan Liu, Qiqin Mo, Pengpeng Liu, Xiao Xiao, Hanhui Ma, Enhancing prime editing efficiency and flexibility with tethered and split pegRNAs, *Protein & Cell*, Volume 14, Issue 4, April 2023, Pages 304–308, https://doi.org/10.1093/procel/pwac014

In the originally published version of this manuscript, the author affiliations were incorrectly assigned. The correct version is:

Ying Feng^1,2,‡^, Siyuan Liu^2,‡,^ Qiqin Mo^2^, Pengpeng Liu^3^, Xiao Xiao^1,*^, Hanhui Ma^2,*^

^1^ School of Biotechnology, East China University of Science and Technology, Shanghai 200237, China

^2^ Gene Editing Center, School of Life Science and Technology, ShanghaiTech University, Shanghai 200135, China

^3^ Department of Molecular, Cell and Cancer Biology, University of Massachusetts Medical School, Worcester, MA 01655, USA

^‡^These authors contributed equally to this work.

^*^Correspondence: mahh@shanghaitech.edu.cn (H. Ma), xiaoxiao@ecust.edu.cn (X. Xiao)

instead of

Ying Feng^1,2,‡^, Siyuan Liu^2,‡,^ Qiqin Mo^2^, Pengpeng Liu^3^, Xiao Xiao^1,*^, Hanhui Ma^2,*^

^1^Gene Editing Center, School of Life Science and Technology, ShanghaiTech University, Shanghai 200135, China

^2^School of Biotechnology, East China University of Science and Technology, Shanghai 200237, China

^3^Department of Molecular, Cell and Cancer Biology, University of Massachusetts Medical School, Worcester, MA 01655, USA

^‡^These authors contributed equally to this work.

^*^Correspondence: mahh@shanghaitech.edu.cn (H. Ma), xiaoxiao@ecust.edu.cn (X. Xiao)

This error has been corrected online. The publisher apologizes for this error.

