# Supplementary material for: Enhancing prime editing efficiency and flexibility with tethered and split pegRNAs
Source: Protein Cell. 2022 Jul 15;14(4):304–8. doi: 10.1093/procel/pwac014 (PMC10120949; doi:10.1093/procel/pwac014)
Supplement: pwac014_suppl_Supplementary_Material [file pwac014_suppl_supplementary_material.pdf]

Spplementary Materials

Materials and methods

Plasmid construction. Modified pegRNAs or split pegRNAs expression plasmids pPB- PE-ALL-RNA, pPB-sPE-ALL-RNA, or pPB-SnPE-ALL-RNA for sPEs tPEs or SnPEs were generated by two steps (Figure S9). First, pPE-pegRNA, psPE-pegRNA, pSnPE- 5'-pRNA, pSnPE-3'-pRNA, pSnPE-circ-pRNA and pSnPE-sgRNA pPE3-sgRNA were constructed separately. For pPE-pegRNA, psPE-pegRNA, pSnPE-5'-pRNA, pSnPE-3'- pRNA and pSnPE-circ-pRNA (Table S1), the oligos were synthesized and annealed with 5' ACCG and 3' AAAA overhangs and cloned into pLH-AAAA backbone plasmid (Sequence S1). For pSnPE-sgRNA and pPE3-sgRNA, the oligos were synthesized and annealed with 5' ACCG and 3' AAAC overhangs and cloned into pLH-sgRNA3 backbone vector (Sequence S1). Second, Golden Gate assembly was used to clone related RNA expression cassettes into one vector pDONOR5.1 (sequence S1) and generate pPB-PE-ALL-RNA, pPB-sPE-ALL-RNA, or pPB-SnPE-ALL-RNA respectively. pCMV-PE2 (#addgene 132775) was used to generate RBP-PE2 (SpCas9H840A-MMLV) expression plasmids. First, T2A-GFP was inserted at the C terminal of pCMV- PE2 resulting in pCMV-PE2-T2A-GFP. Second, RNA binding proteins for stem loop aptamers including MCP, tdMCP, PCP, tdPCP, Com, N22p and Csy4H29A (Sequence S2) were synthesized and cloned to the pCMV-PE2-T2A-GFP using ClonExpress II One Step Cloning Kit (Vazyme, C112-01), resulting pCMV-MCP-PE2-T2A-GFP, pCMV- tdMCP-PE2-T2A-GFP, pCMV-PCP-PE2-T2A-GFP, pCMV-tdPCP-PE2-T2A-GFP, pCMV-Com-PE2-T2A-GFP, pCMV-N22p-PE2-T2A-GFP and pCMV-Csy4H29A-PE2- T2A-GFP respectively.

Cell culture and transfection and genomic DNA extraction. HEK293FT (Thermo Fisher Scientific), HeLa (ATCC, CCL-2), and U2OS (ATCC, HTB-96) cells were cultured in DMEM with high glucose in 10% FBS (fetal bovine serum, Thermo Fisher Scientific). All cells were cultured at 37°C and 5% CO_2 in a humidified incubator. Cells were seeded in 12-well plates and transfected at approximately 60% confluence using Lipofectamine 2000 (Thermo Fisher Scientific) according to the manufacturer's protocols. A total of 1.5 µg Prime editor 2 (PE2, dCas9-MMLV) or RBP-PE2, and 500 ng PE-all-RNA, sPE-all- RNA, tPE-all-RNA or snPE-all-RNA expression plasmids were co-transfected into HEK293FT, U2OS or HeLa cells. 72 h after

transfection, Prime editors (GFP+) and PE- all-RNA, sPE-all-RNA, tPE-all-RNA or snPE-all-RNA (BFP+) double-positive cells were collected from flow cytometry (BD FACS Aria III). The genomic DNA of cells was extracted using QuickExtract DNA Extraction Solution (Lucigen) according to the manufacturer's protocols. The isolated DNA was PCR-amplified with Phanta Max Super-Fidelity DNA Polymerase (Vazyme). Primers used are listed in Table S2.

High-throughput DNA sequencing of genomic DNA samples. Genomic sites of interest were amplified from genomic DNA using locus-specific primers containing the Truseq adapters. Equal amounts of PCR products were pooled and gel purified. The purified library was deep sequenced using a paired-end 150 bp Illumina MiniSeq run. All prime editing experiments were analyzed as follows. Demultiplexing and base calling were both performed using bcl2fastq Conversion Software v2.18 (Illumina, Inc.), allowing 0 barcode mismatches with a minimum trimmed read length of 75. Alignment of sequencing reads to the Amplicon sequence (Table S5) was performed using CRISPResso2 (Clement et al., 2019) in standard mode using the parameters ''-q 30''. For each amplicon, the CRISPResso2 quantification window was positioned to include the entire sequence between pegRNA- and sgRNA-directed Cas9 cut sites, as well as an additional 10 bp beyond both cut sites. For PE activity quantifications from deep sequenced data, editing efficiency was calculated as the percentage of reads with the desired editing without indels (''-discard_indel_reads TRUE.'' mode) out of the total number of reads ((number of desired editing-containing reads)/(number of reference- aligned reads).). For all experiments, indel frequency was calculated as the number of discarded reads divided by the total number of reads ((number of indel-containing reads)/(number of reference-aligned reads). EditR (Kluesner et al., 2018) was used for PE activity quantifications from all sanger sequencing data.

Live cell CRISPR-based DNA imaging. All live-cell imaging was carried out on a DeltaVision Ultra imaging system (GE Healthcare). The cells were cultured on No. 1.0 glass bottom dishes (MatTek). The microscope stage incubation chamber was maintained at 37°C and 5% CO2. GFP was excited at 488 nm and collected using filter at 498/30 nm (wavelength/bandwidth). Imaging data were acquired by DeltaVision Elite imaging (GE Healthcare Inc.) software. For the representative images, the raw data were deconvoluted by softWoRx (GE Healthcare Inc.) software.

Off-target analysis. Potential off-target sites were predicted in the human genome (GRCh38/hg38) with Cas-OFFinder6 (http://www.rgenome.net/cas-offinder); The region around the off-target sites was amplified with Phanta Max Super-Fidelity DNA Polymerase (Vazyme), and subjected to high-throughput sequencing. The amplicons were analyzed with CRIPResso2 (V2.0.43) and the off-target sites are listed in Supplementary Table 3. Primers used are listed in Supplementary Table 4.

Data availability

The next-generation sequencing data have been deposited in the NCBI Sequence Read Archive database under the BioProject accession code PRJNA785066. All other relevant data are available from corresponding authors upon reasonable request.

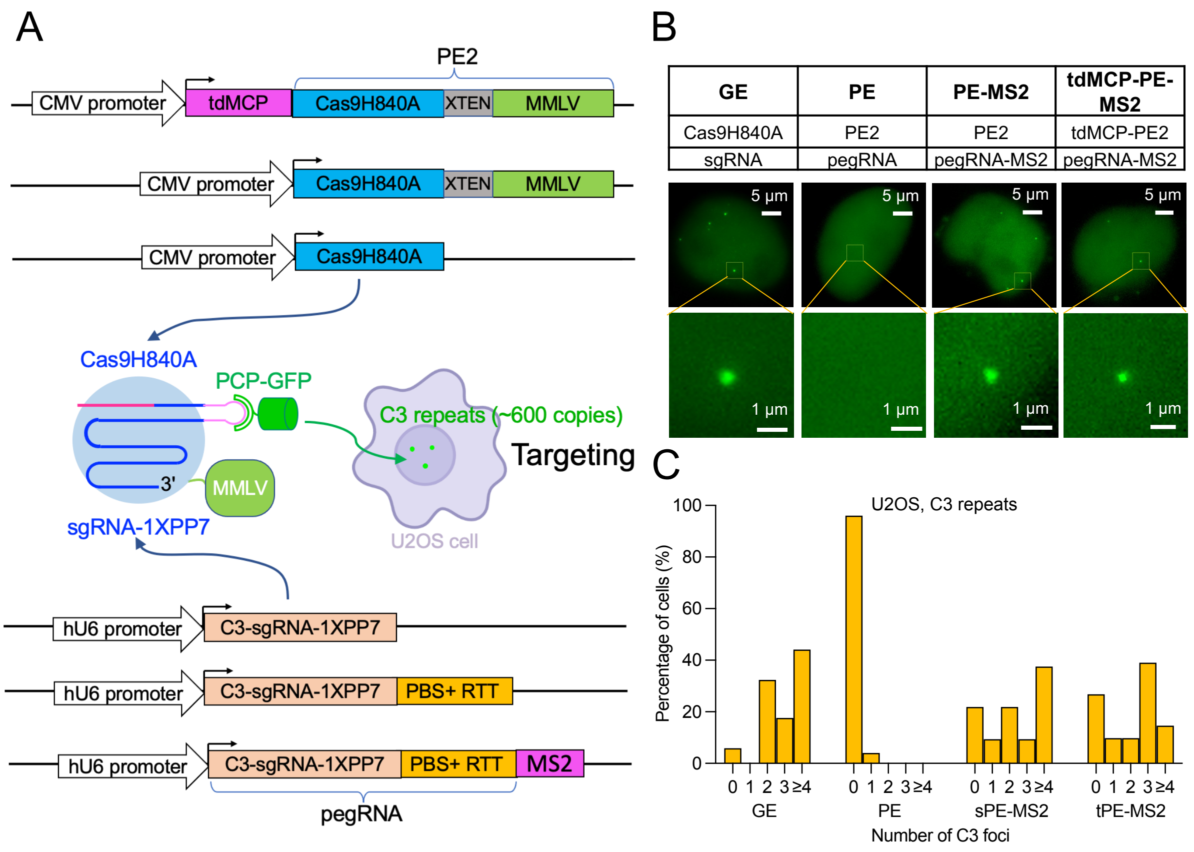

Figure S1 | Increase of targeting efficiency by adding stem-loop RNA aptamers at the 3'-terminal of pegRNAs.

(A) Schematic of the live-cell detection system for targeting efficiency. The detection system consists of Cas9 nickase, PCP-GFP, sgRNA-1XPP7 targeting C3 repeats (~600 copies of target sites on chromosome 3). The expression of Cas9H840A, Cas9H840A-MMLV (PE2) or tdMCP-PE2 was driven by CMV promoter. The expression of sgRNA, pegRNA or pegRNA-MS2 was driven by human U6 (hU6) promoter. (B) U2OS cells were co-transfected PCP-GFP along with expression plasmids as indicated for GE, PE, sPE-MS2, or tPE-MS2. After 48 hours, cells were imaged by collecting z-stack images to capture all foci in each nucleus examined. Representative images show the 2D projection of 3D imaging. (C) Histograms showing the number of C3 foci per cell counts by CRISPR-based labeling, n=50 transfected cells of GE, PE, sPE-MS2 or tPE-MS2.

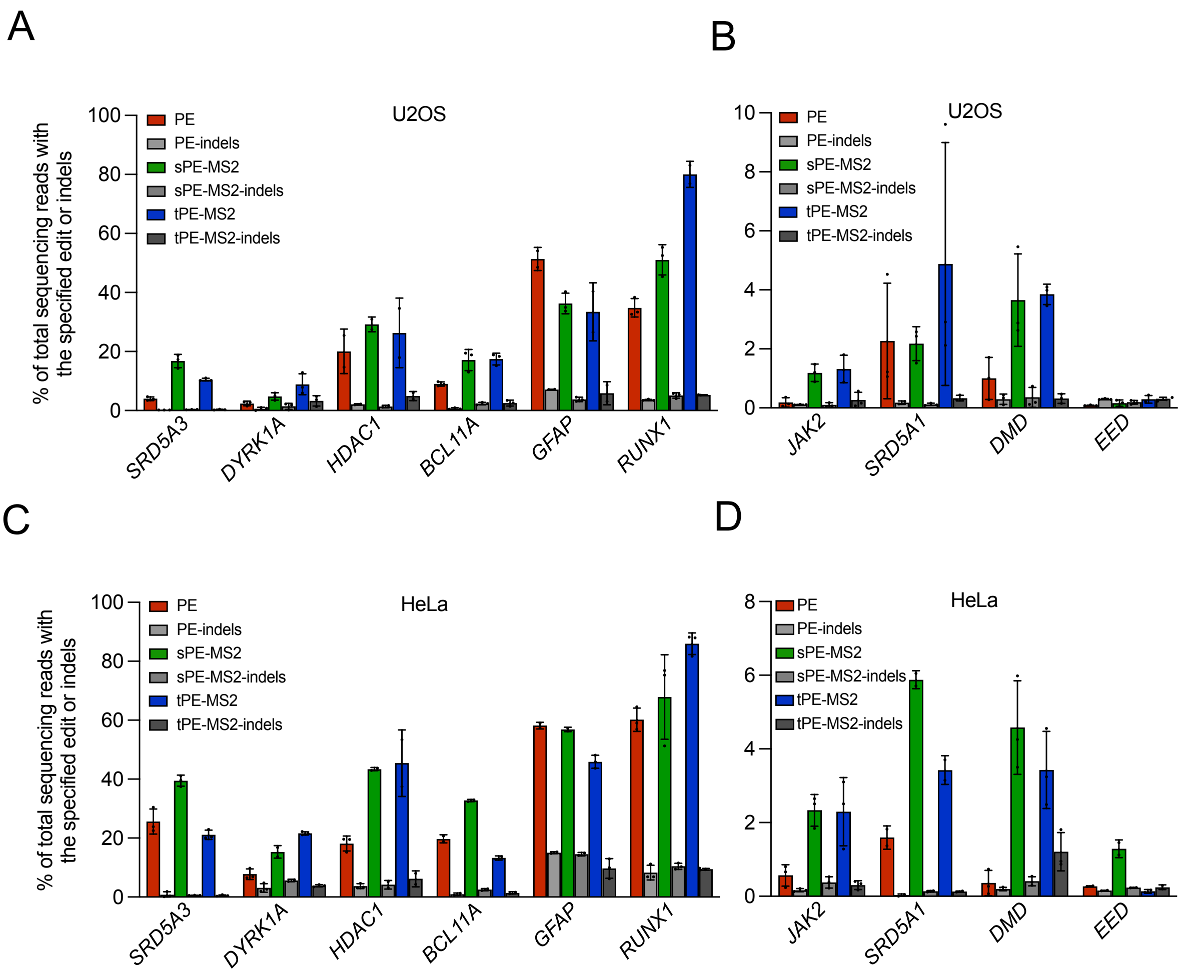

Figure S2 | sPEs and tPEs increase PE efficiency at various loci in different cell lines.

Efficiency of PE, sPE-MS2 and tPEb-MS2 mediated point mutation of *SRD5A3*_+2 C·G to A·T, *DYRK1A*_+1 C·G to G·C, *HDAC1*_+1 C·G to G·C, *BCL11A*_+1C·G to A·T, *GFAP*_+1A·T to T·A and *RUNX1*_+5 G·C to T·A using PE3 in U2OS cells (A), Hela cells (C). Efficiency of PE, sPE-MS2 and tPE-MS2 mediated point mutation of *DMD*_+1 T·A to C·G, *EED*_+1 A·T to T·A, *JAK2* _+1 C·G to T·A and *SRD5A1*_+1 C·G to A·T using PE3 in U2OS cells (B), Hela cells (D). Data and error bars in (A-D) indicate the mean and standard deviation of three independent biological replicates.

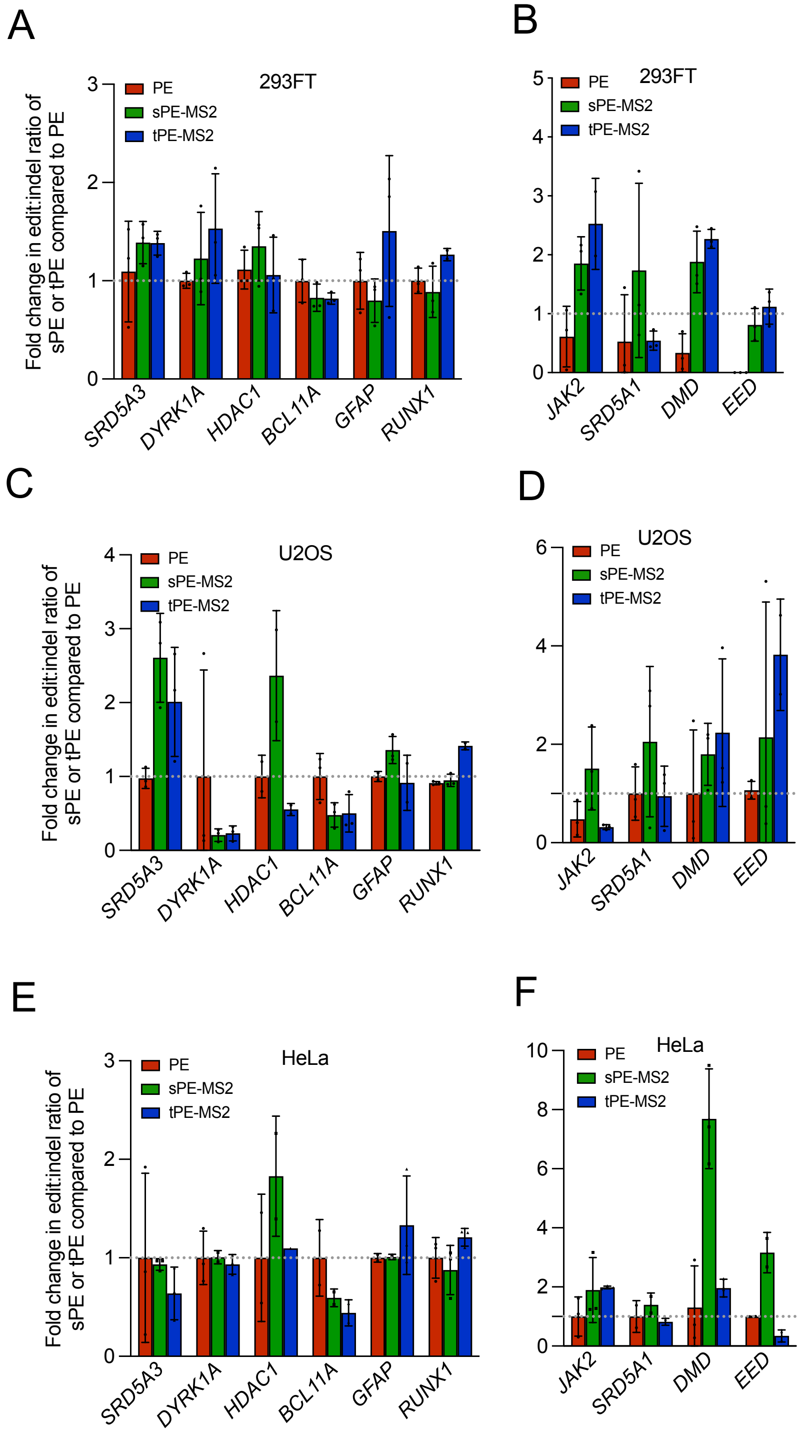

Figure S3 | Fold change in correct editing and Edit:indel ratio for sPE-MS2 and tPE-MS2 at different loci shown in Figure 1 and Figure S2.

Fold-change in the observed edit:indel ratio of PE, sPE-MS2 and tPE-MS2 mediated point mutation for *SRD5A3*_+2 C·G to A·T, *DYRK1A*_+1 C·G to G·C, *HDAC1*_+1 C·G

to G·C, *BCL11A*_+1C·G to A·T, *GFAP*_+1A·T to T·A and *RUNX1*_+5 G·C to T·A in HEK293FT cells (A) U2OS cells (C) and HeLa cells (E). Fold-change in the observed edit:indel ratio of PE, sPE-MS2 and tPEb-MS2 mediated point mutation of *DMD*_+1 T·A to C·G, *EED*_+1 A·T to T·A, *JAK2*_+1 C·G to T·A and *SRD5A1*_+1 C·G to A·T in HEK293FT cells (B) U2OS cells (D) and HeLa cells (F). Values were calculated from the data presented in Figure 1 and Figure S2. Data and error bars indicate the mean and standard deviation of three in dependent biological replicates.

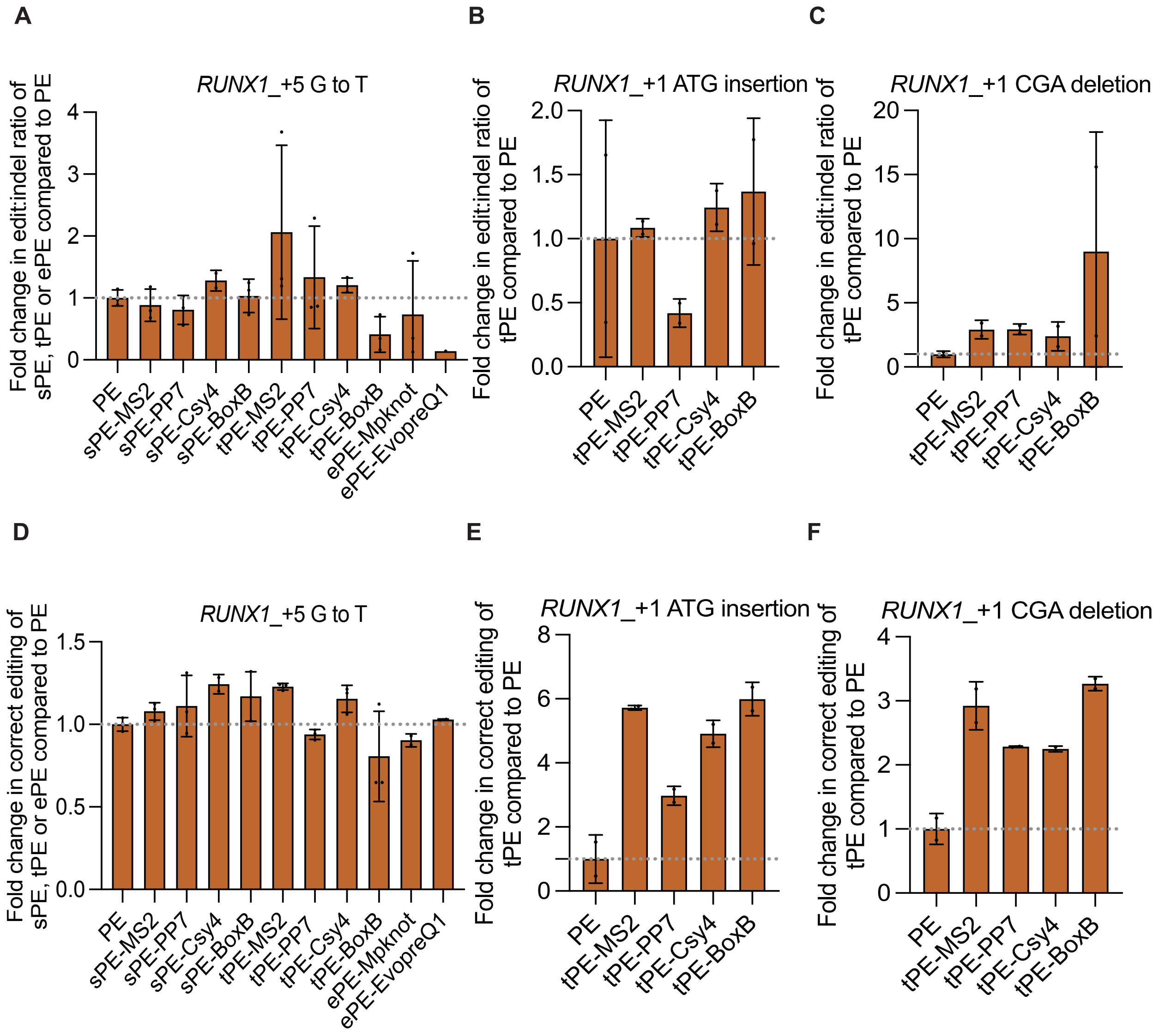

Figure S4 | Fold change in correct editing and edit:indel ratio for sPEs and tPEs shown in Figure 1.

(A) Fold-change in the observed edit:indel ratio of PE, sPE-MS2, tPE-MS2, ePE-Mpknot and ePE-EvopreQ1 mediated with point mutation for *RUNX1*_+5 G·C to T·A in HEK293FT cells. (B) Fold-change in the observed edit:indel ratio of PE, tPE-MS2, tPE-PP7, tPE-Csy4 and tPE-BoxB mediated with insertion for *RUNX1*_+1 ATG in HEK293FT cells. (C) Fold-change in the observed edit:indel ratio of PE, tPE-MS2, tPE-PP7, tPE-Csy4 and tPE-BoxB mediated with deletion for *RUNX1*_+1 CGA in HEK293FT cells. (D) Fold-change in the observed correct editing of PE, sPE-MS2, tPE-MS2, ePE-Mpknot and ePE-EvopreQ1 mediated with point mutation for *RUNX1*_+5 G·C to T·A in

HEK293FT cells. (E) Fold-change in the observed correct editing of PE, tPE-MS2, tPE-PP7, tPE-Csy4 and tPE-BoxB mediated with insertion for *RUNX1*_+1 ATG in HEK293FT cells. (F) Fold-change in the observed correct editing of PE, tPE-MS2, tPE-PP7, tPE-Csy4 and tPE-BoxB mediated with deletion for *RUNX1*_+1 CGA in HEK293FT cells. Values were calculated from the data presented in Figure 1. Data and error bars indicate the mean and standard deviation of three in dependent biological replicates.

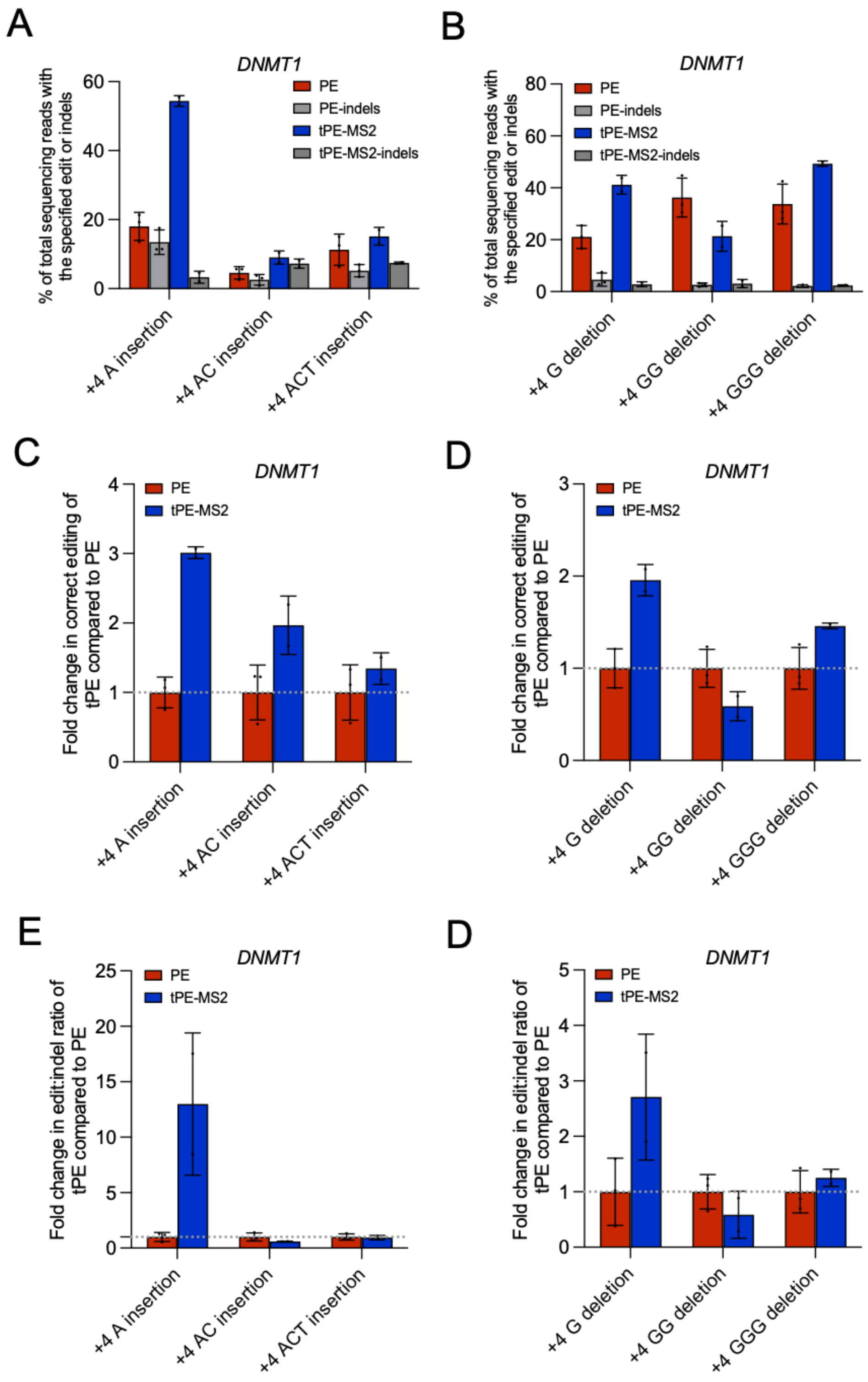

Figure S5 | Insertion and deletion efficiency for *DNMT1* loci and fold change in correct editing and edit:indel ratio.

(A) The efficiency of PE and tPE-MS2 mediated insertion of DNMT1_+4 A, +4 AC, +4 ACT using PE3 in 293FT cells. (B) The efficiency of PE and tPE-MS2 mediated deletion of DNMT1_+4 G, +4 GG, +4 GGG using PE3 in HEK293FT cells. (C) Fold-change in the observed correct editing ratio of PE, tPE-MS2 mediated with insertion for *DNMT1*_+4 A, *DNMT1*_+4 AC, *DNMT1*_+4ACT in HEK293FT cells. (D) Fold-change in the observed correct editing ratio of PE, tPE-MS2 mediated with deletion for *DNMT1*_+4 G, *DNMT1*_+4 GG, *DNMT1*_+4GGG in HEK293FT cells. (E) Fold-change in the observed edit:indel ratio of PE, tPE-MS2 mediated with insertion for *DNMT1*_+4 A, *DNMT1*_+4 AC, *DNMT1*_+4ACT in HEK293FT cells. (F) Fold-change in the observed edit:indel ratio of PE, tPE-MS2 mediated with deletion for *DNMT1*_+4 G, *DNMT1*_+4 GG, *DNMT1*_+4GGG in HEK293FT cells. Values were calculated from the data presented in Figure S5A and Figure S5B. Data and error bars indicate the mean and standard deviation of three in dependent biological replicates.

DYRK1A	PE	sPE-MS2	tPE-MS2
On target	8.0 (1.5)	20.6 (3.7)	28.3 (4.8)
Off-target site 1	<0.1 (<0.7)	<0.1 (2.4)	<0.1 (0.7)
Off-target site 2	<0.1 (<0.3)	<0.1 (<0.7)	<0.1 (0.1)

RUNX1	PE	sPE-MS2	tPE-MS2
On target	70.3 (1.1)	75.9 (1.1)	83.9 (<0.7)
Off-target site 1	<0.1 (5.3)	<0.1 (2.8)	<0.1 (1.0)
Off-target site 2	<0.1 (<0.3)	<0.1 (<0.3)	<0.1 (<0.3)
Off-target site 3	<0.1 (<0.7)	<0.1 (<0.1)	<0.1 (<0.3)

BCL11A	PE	sPE-MS2	tPE-MS2
On target	22.6 (5.9)	34.1 (14.5)	26.6 (10.9)
Off-target site 1	<0.1 (<0.3)	<0.1 (<0.3)	<0.1 (<0.3)
Off-target site 2	<0.1 (<0.1)	<0.1 (<0.1)	<0.1 (<0.3)

SRD5A3	PE	sPE-MS2	tPE-MS2
On target	10.2 (<0.7)	17.3 (<0.7)	12.4 (<0.7)
Off-target site 1	<0.1 (0.7)	<0.1 (<0.3)	<0.1 (<0.3)
Off-target site 2	<0.1 (<0.3)	<0.1 (<0.3)	<0.1 (<0.3)
Off-target site 3	<0.1 (<0.1)	<0.1 (<0.3)	<0.1 (<0.3)
Off-target site 4	<0.1 (<0.1)	<0.1 (<0.3)	<0.1 (<0.3)

JAK2	PE	sPE-MS2	tPE-MS2
On target	1.3 (<0.3)	4.1 (<0.3)	5.5 (<0.3)
Off-target site 1	<0.1 (<0.3)	<0.1 (<0.3)	<0.1 (<0.3)
Off-target site 2	<0.1 (<0.3)	<0.1 (<0.3)	<0.1 (<0.3)
Off-target site 3	<0.1 (<0.3)	<0.1 (<0.1)	<0.1 (<0.3)

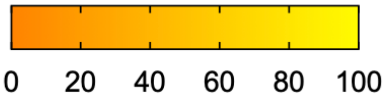

Figure S6 | Comparison of off-target activity between canonical PE, sPE-MS2 and tPE-MS2. on-target and off-target activities of PE, sPE-MS2 and tPE-MS2 at *DYRK1A*, *RUNX1*, *BCL11A*, *SRD5A3* and *JAK2* were detected. PE, sPE and tPE editing is shown as % prime editing alongside % indels (in parentheses).

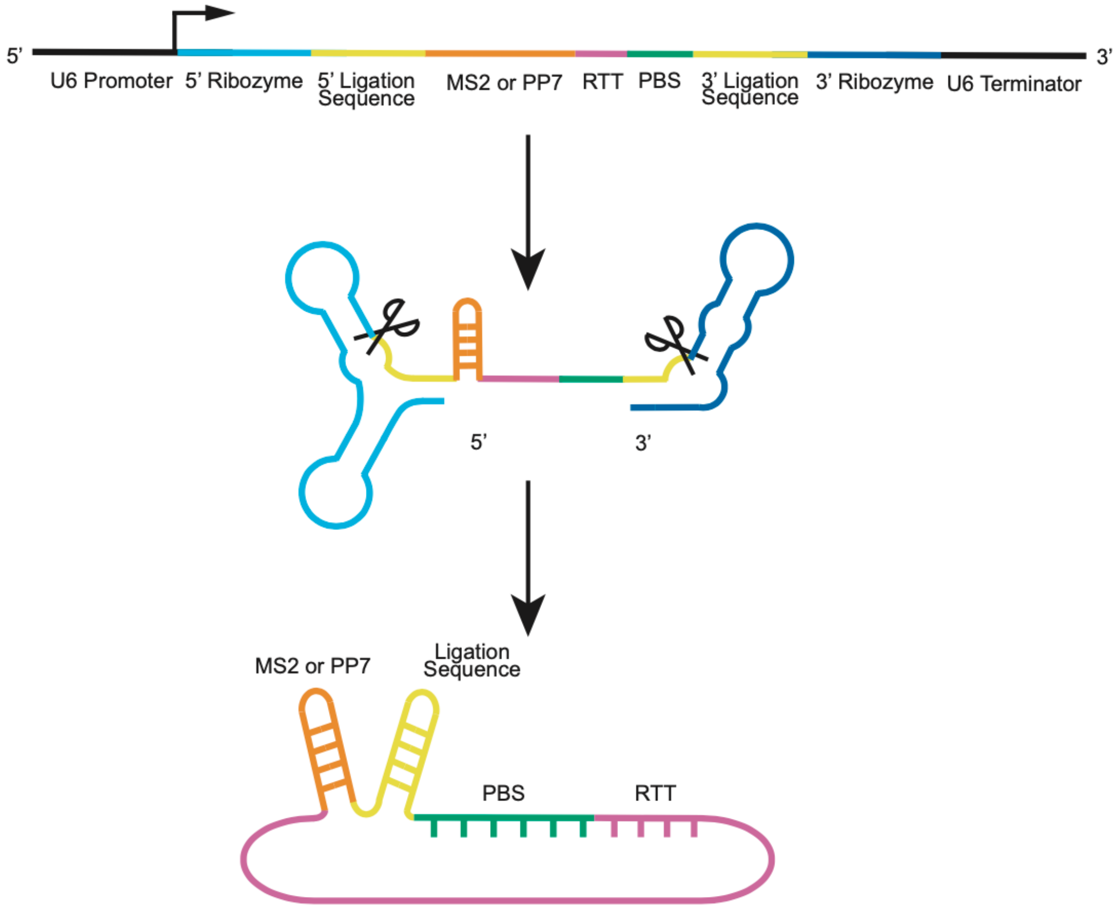

Figure S7 | Circular prime RNA generated by the Tornado expression system used in Figure 2.

Construct design for generating autocatalytically processed circular prime RNA (cpRNA) by the Tornado expression system. The circular prime RNA in the Tornado cassette is driven by U6 promoter. The circular pRNA consists of MS2 or PP7 (orange), RTT (reddish-purple) and PBS (bluish-green). The circular pRNA is flanked by the 5′- and 3′-stem-forming sequences (yellow), each of which is flanked by the 5′- and 3′-self-cleaving ribozymes (sky blue and blue, respectively). The stem-forming by 5’- and 3’-ligation sequences facilitates the circularization of pRNA into circular prime RNA (cpRNA).

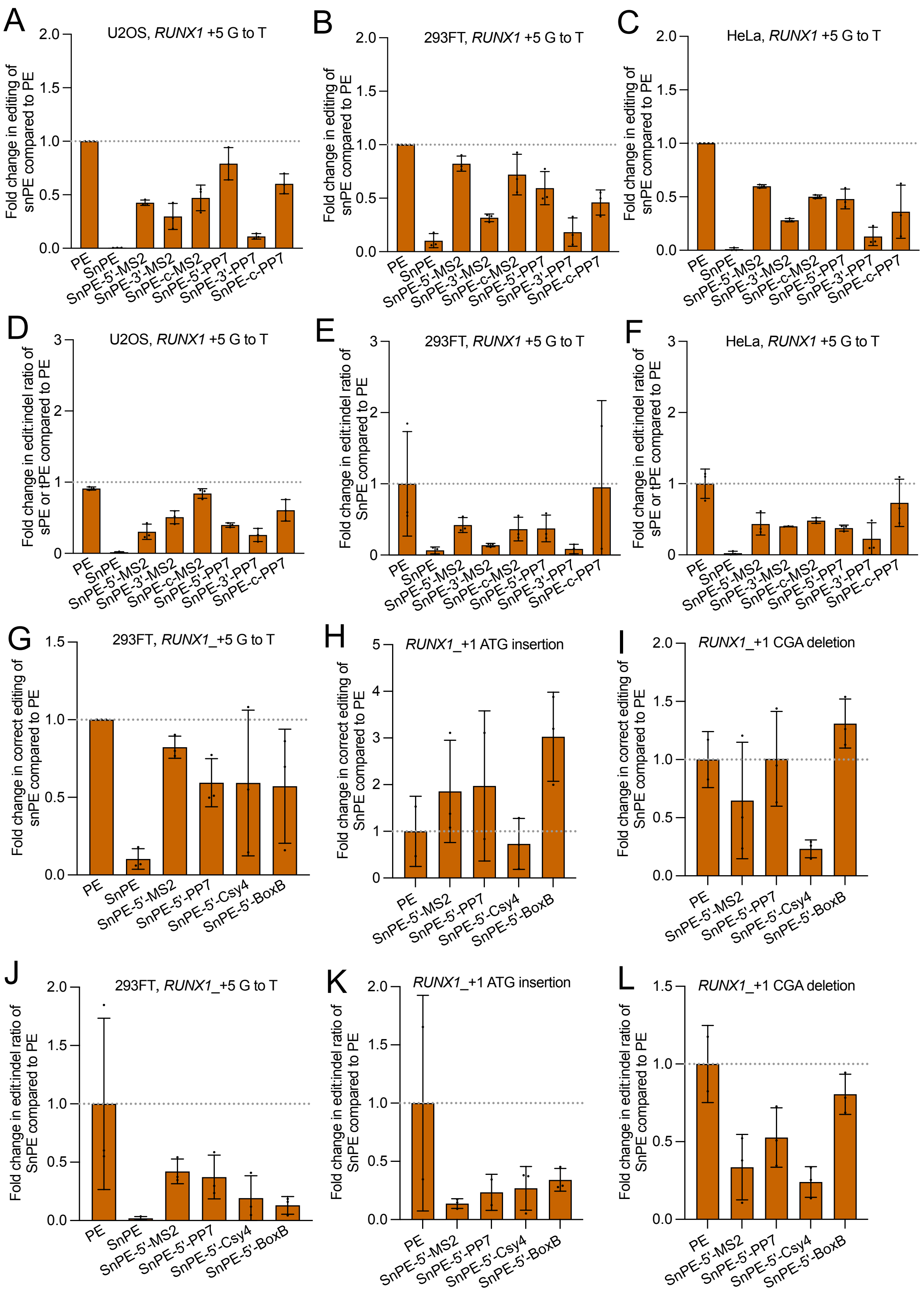

A
U2OS, RUNX1 +5 G to T
Fold change in editing of snPE compared to PE
B
293FT, RUNX1 +5 G to T
Fold change in editing of snPE compared to PE
C
HeLa, RUNX1 +5 G to T
Fold change in editing of snPE compared to PE
PE
SnPE
SnPE-5'-MS2
SnPE-3'-MS2
SnPE-c-MS2
SnPE-5'-PP7
SnPE-3'-PP7
SnPE-c-PP7
D
U2OS, RUNX1 +5 G to T
Fold change in edit:indel ratio of sPE or tPE compared to PE
E
293FT, RUNX1 +5 G to T
Fold change in edit:indel ratio of SnPE compared to PE
F
HeLa, RUNX1 +5 G to T
Fold change in edit:indel ratio of sPE or tPE compared to PE
G
293FT, RUNX1_+5 G to T
Fold change in correct editing of snPE compared to PE
H
RUNX1_+1 ATG insertion
Fold change in correct editing of SnPE compared to PE
I
RUNX1_+1 CGA deletion
Fold change in correct editing of SnPE compared to PE
SnPE-5'-Csy4
SnPE-5'-BoxB
J
293FT, RUNX1_+5 G to T
Fold change in edit:indel ratio of SnPE compared to PE
K
RUNX1_+1 ATG insertion
Fold change in edit:indel ratio of SnPE compared to PE
L
RUNX1_+1 CGA deletion
Fold change in edit:indel ratio of SnPE compared to PE

Figure S8 | Fold change in correct editing and edit:indel ratio for SnPEs shown in Figure 2.

Fold-change in the observed correct editing for *RUNX1*_+5 G·C to T·A transversion of SnPE-5'-MS2 or PP7, SnPE-3'-MS2 or PP7, SnPE-c-MS2 or PP7, compared to canonical PE (dashed line) in U2OS cells (A), HEK293FT cells (B), and HeLa cells (C). Fold-change in the observed edit:indel ratio for *RUNX1*_+5 G·C to T·A of SnPE-5'-MS2 or PP7, SnPE-3'-MS2 or PP7, SnPE-c-MS2 or PP7, compared to canonical PE (dashed line) in U2OS cells (D), HEK293FT cells (E), and HeLa cells (F). (G) Fold-change in the observed correct edting for SnPE-5'-MS2, SnPE-5'-PP7, SnPE-5'-Csy4 and SnPE-5'-boxB mediated with *RUNX1*_+5 G·C to T·A in HEK293FT cells. (H) Fold-change in the observed correct editing of PE, tPE-MS2, tPE-PP7, tPE-Csy4 and tPE-BoxB mediated with insertion for *RUNX1*_+1 ATG in HEK293FT cells. (I) Fold-change in the observed correct editing of PE, tPE-MS2, tPE-PP7, tPE-Csy4 and tPE-BoxB mediated with deletion for *RUNX1*_+1 CGA in HEK293FT cells. (J) Fold-change in the observed edit:indel ratio for SnPE-5'-MS2, SnPE-5'-PP7, SnPE-5'-Csy4 and SnPE-5'-boxB mediated with *RUNX1*_+5 G·C to T·A in HEK293FT cells. (K) Fold-change in the observed edit:indel ratio of PE, tPE-MS2, tPE-PP7, tPE-Csy4 and tPE-BoxB mediated with insertion for *RUNX1*_+1 ATG in HEK293FT cells. (L) Fold-change in the observed edit:indel ratio of PE, tPE-MS2, tPE-PP7, tPE-Csy4 and tPE-BoxB mediated with deletion for *RUNX1*_+1 CGA in HEK293FT cells. Values were calculated from the data presented in Figure 2. Data and error bars reflect the mean and standard deviation of three independent biological replicates.

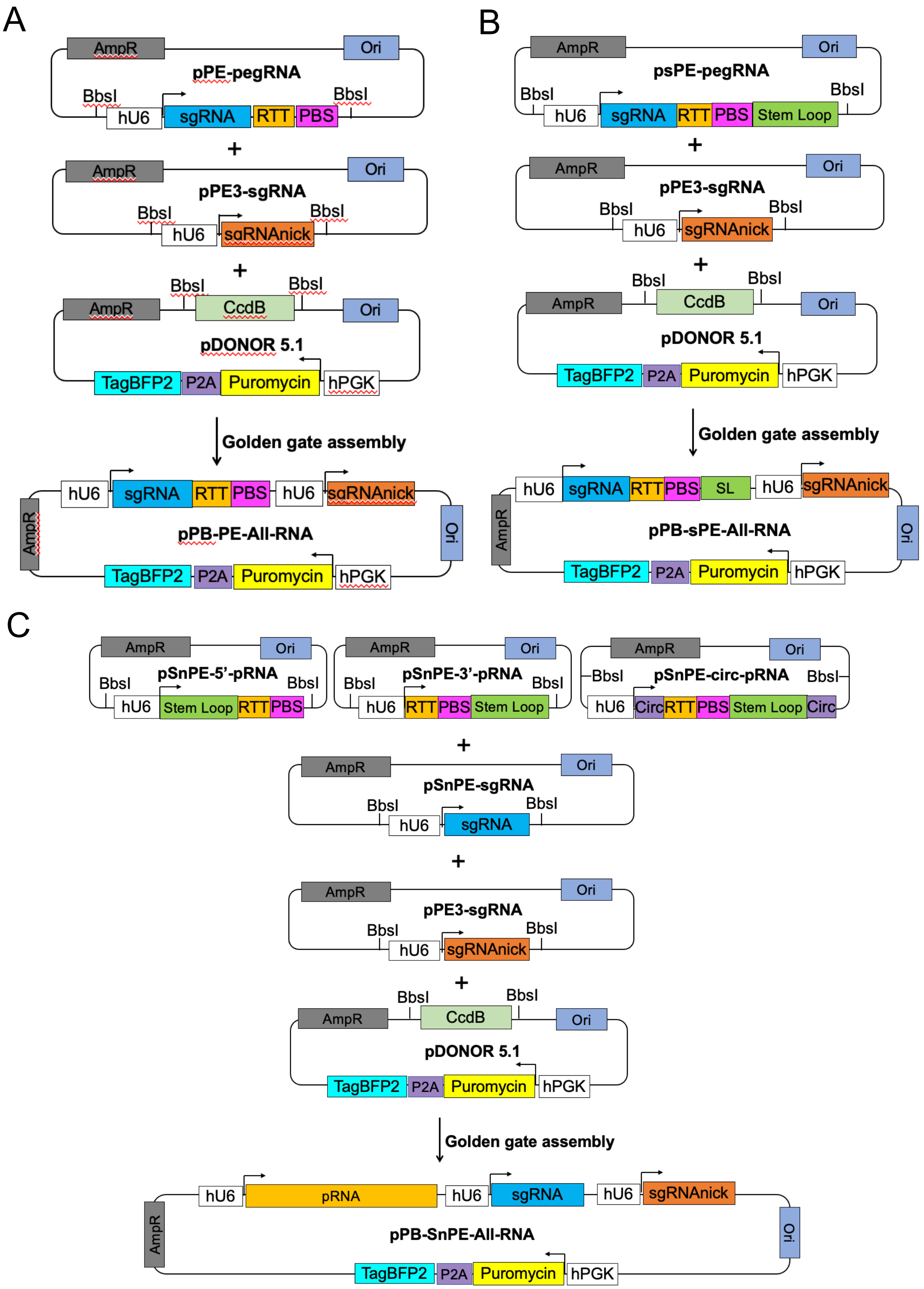
A
AmpR
Ori
pPE-pegRNA
BbsI
hU6
sgRNA
RTT
PBS
+
pPE3-sgRNA
sgRNAnick
CcdB
pDONOR 5.1
TagBFP2
P2A
Puromycin
hPGK
Golden gate assembly
pPB-PE-All-RNA
B
psPE-pegRNA
Stem Loop
SL
pPB-sPE-All-RNA
C
pSnPE-5'-pRNA
pSnPE-3'-pRNA
pSnPE-circ-pRNA
Circ
pSnPE-sgRNA
pRNA
pPB-SnPE-All-RNA

Figure S9 | Cloning strategy for single construct containing all RNA expression components for modified or split pegRNA prime editors.

Construction of pPB-PE-ALL-RNA for canonical PE, pPB-sPE-ALL-RNA plasmids for sPEs and tPEs, and pPB-SnPE-ALL-RNA for SnPEs. (A) pPB-PE-ALL-RNA was reconstituted from pPE-pegRNA, pPE3-sgRNA and pDONOR5.1 by golden gate assembly, pPE-pegRNA consists of sgRNA (blue), RTT (gold) and PBS (Magenta), pPE3-sgRNA contains sgRNAnick (orange), pDONOR5.1 consists of CcdB gene (green), Puromycin (yellow), P2A (purple) and TagBFP2 (Cyan). (B) pPB-sPE-ALL-RNA was reconstituted from psPE-pegRNA, which contains sgRNA (blue), RTT (gold), PBS (Magenta) and stem-loop RNA aptamer (dark green), pPE3-sgRNA and pDONOR5.1 were the same in (A). (C) pPB-SnPE-ALL-RNA contains three types of pRNA: pSnPE-5'-pRNA, pSnPE-3'-pRNA and pSnPE-circ-pRNA. pSnPE-5'-pRNA consists of stem loop at 5'-terminal (dark green), RTT (gold) and PBS (Magenta). pSnPE-3'-pRNA contains stem-loop at 3'-terminal (dark green), RTT (gold) and PBS (Magenta). pSnPE-circ-pRNA contains circular sequence located at both terminals (purple), stem loop (dark green), RTT (gold) and PBS (Magenta). pSnPE-sgRNA was pSnPE-pRNAs, sgRNA (blue), pPE3-sgRNA and pDONOR5.1 that were used in (A) and (B).

Supplementary Table 1 | Sequences of primers used for pegRNA cloning

pPE-pegRNA	Forward	Reverse
JAK2	ataattgaagacATaccgACCTGTGACTCATGAAACACgtttgagagctagaaatagcaagttcaaataaggc	ataattgaagacGCaaaaCTGTGACTCATGAAATACAGGAAGAATGTgcaccgactcggtgcc
HDAC1	ataattgaagacATaccgGCACCATGCAAAGAAGTCCGgtttgagagctagaaatagcaagttcaaataaggc	ataattgaagacGCaaaaTGCAAAGAAGTGCGAGGCATCTGGCTgcaccgactcggtgcc
EED	ataattgaagacATaccgCCGGTTGTTGCAATCTTACGgtttgagagctagaaatagcaagttcaaataaggc	ataattgaagacGCaaaaGTTGTTGCAATCTTTCGTGGATGCTGATgcaccgactcggtgcc
BCL11A	ataattgaagacATaccgtcacagATAAACTTCTGCACgtttgagagctagaaatagcaagttcaaataaggc	ataattgaagacGCaaaaCACAGATAAACTTCTGAACTGGAGGGGCCgcaccgactcggtgcc
SRD5A3	ataattgaagacATaccgCCTTACTCAATCTCTGTTCCgtttgagagctagaaatagcaagttcaaataaggc	ataattgaagacGCaaaaTTACTCAATCTCTGTTACTGGGAGCACCTTTTCgcaccgactcggtgcc
SRD5A1	ataattgaagacATaccgTCCATTCAGATCATATCCTAgtttgagagctagaaatagcaagttcaaataaggc	ataattgaagacGCaaaaCATTCAGATCATATCATAAGGAATCTCAGAAAgcaccgactcggtgcc
DYRK1A	ataattgaagacATaccgagcacatcaaggacattctagtttgagagctagaaatagcaagttcaaataaggc	ataattgaagacGCaaaacaaggacattgtaaggatgatgcaccgactcggtgcc
DMD	ataattgaagacATaccgCTACCTCTGTGATACTCTTCgtttgagagctagaaatagcaagttcaaataaggc	ataattgaagacGCaaaaACCTCTGTGATACTCCTCAGGTGCACCTgcaccgactcggtgcc
RUNX1	ataattgaagacATaccggcattttcaggaggaagcgagtttgagagctagaaatagcaagttcaaataaggc	ataattgaagacGCaaaaattttcaggaggaagcgattgcttcagacagcaccgactcggtgcc
GFAP	ataattgaagacATaccgagcctgtgtccatataaagggttttagagctagaaatagcaagttaaaataaggctagtc	ataattgaagacGCaaaaTGTGTCCATATAATGGAGGAGTTGGAAgcaccgactcggtgcc
psPE-pegRNA	Forward	Reverse
JAK2-MS2	ataattgaagacATaccgACCTGTGACTCATGAAACACgtttgagagctagaaatagcaagttcaaataaggc	ataattgaagacGCaaaaacatgggtgatcctcatgtCTGTGACTCATGAAATACAGGAAGAATGTgcaccgactcggtgcc
HDAC1-MS2	ataattgaagacATaccgGCACCATGCAAAGAAGTCCGgtttgagagctagaaatagcaagttcaaataaggc	ataattgaagacGCaaaaacatgggtgatcctcatgtTGCAAAGAAGTGCGAGGCATCTGGCTgcaccgactcggtgcc
EED-MS2	ataattgaagacATaccgCCGGTTGTTGCAATCTTACGgtttgagagctagaaatagcaagttcaaataaggc	ataattgaagacGCaaaaacatgggtgatcctcatgtGTTGTTGCAATCTTTCGTGGATGCTGATgcaccgactcggtgcc
BCL11A-MS2	ataattgaagacATaccgtcacagATAAACTTCTGCACgtttgagagctagaaatagcaagttcaaataaggc	ataattgaagacGCaaaaacatgggtgatcctcatgtCACAGATAAACTTCTGAACTGGAGGGGCCgcaccgactcggtgcc
SRD5A3-MS2	ataattgaagacATaccgCCTTACTCAATCTCTGTTCCgtttgagagctagaaatagcaagttcaaataaggc	ataattgaagacGCaaaaacatgggtgatcctcatgtTTACTCAATCTCTGTTACTGGGAGCACCTTTTCgcaccgactcggtgcc
SRD5A1-MS2	ataattgaagacATaccgTCCATTCAGATCATATCCTAgtttgagagctagaaatagcaagttcaaataaggc	ataattgaagacGCaaaaacatgggtgatcctcatgtCATTCAGATCATATCATAAGGAATCTCAGAAAgcaccgactcggtgcc
PCSK9-MS2	ataattgaagacATaccgcccgcacCTTGGCGCAGCGGgtttgagagctagaaatagcaagttcaaataaggc	ataattgaagacGCaaaaacatgggtgatcctcatgtTGGCGCAGAGGTGGAAGGTGGCgcaccgactcggtgcc
DYRK1A-MS2	ataattgaagacATaccgagcacatcaaggacattctagtttgagagctagaaatagcaagttcaaataaggc	ataattgaagacGCaaaaacatgggtgatcctcatgtcaaggacattgtaaggatgatgcaccgactcggtgcc
DMD-MS2	ataattgaagacATaccgCTACCTCTGTGATACTCTTCgtttgagagctagaaatagcaagttcaaataaggc	ataattgaagacGCaaaaacatgggtgatcctcatgtACCTCTGTGATACTCCTCAGGTGCACCTgcaccgactcggtgcc
RUNX1-MS2	ataattgaagacATaccggcattttcaggaggaagcgagtttgagagctagaaatagcaagttcaaataaggc	ataattgaagacGCaaaaacatgggtgatcctcatgtattttcaggaggaagcgattgcttcagacagcaccgactcggtgcc
DNMT1-MS2	ataattgaagacATaccgGATTCCTGGTGCCAGAAACAgtttgagagctagaaatagcaagttcaaataaggc	ataattgaagacGCaaaaacatgggtgatcctcatgtCCTGGTGCCAGAAACAAGGGTGACgcaccgactcggtgcc
GFAP-MS2	ataattgaagacATaccgagcctgtgtccatataaagggttttagagctagaaatagcaagttaaaataaggctagtc	ataattgaagacGCaaaaacatgggtgatcctcatgtTGTGTCCATATAATGGAGGAGTTGGAAgcaccgactcggtgcc
RUNX1-com	ataattgaagacATaccggcattttcaggaggaagcgagtttgagagctagaaatagcaagttcaaataaggc	ataattgaagacGCaaaaGATGCTCGCAGGCATTCAGattttcaggaggaagcgattgcttcagacagcaccgactcggtgcc
RUNX1-pp7	ataattgaagacATaccggcattttcaggaggaagcgagtttgagagctagaaatagcaagttcaaataaggc	ataattgaagacGCaaaaggagcgacgccatatcgtctgctccattttcaggaggaagcgattgcttcagacagcaccgactcggtgcc
RUNX1-csy4	ataattgaagacATaccggcattttcaggaggaagcgagtttgagagctagaaatagcaagttcaaataaggc	ataattgaagacGCaaaaCTGCCTATACGGCAGTGAACattttcaggaggaagcgattgcttcagacagcaccgactcggtgcc
RUNX1-boxB	ataattgaagacATaccggcattttcaggaggaagcgagtttgagagctagaaatagcaagttcaaataaggc	ataattgaagacGCaaaaggcccttcttcagggccattttcaggaggaagcgattgcttcagacagcaccgactcggtgcc
pPE3-sgRNA	Forward	Reverse
JAK2	accgTGGGGTTTGATCGTTTTCTT	aaacAAGAAAACGATCAAACCCCA
HDAC1	accgATACTTTAGCAGTTCCAGGA	aaacTCCTGGAACTGCTAAAGTAT
EED	accgTGATTAATTTATAAAAGTTT	aaacAAACTTTTATAAATTAATCA
BCL11A	accgCTGCACTCATCCCAGGCGTG	aaacCACGCCTGGGATGAGTGCAG
SRD5A3	accgCCAGTAACAGAGATTGAGTA	aaacTACTCAATCTCTGTTACTGG
SRD5A1	accgTCCTTATGATATGATCTGAA	aaacTTCAGATCATATCATAAGGA
DYRK1A	accgAAGAGTGAGGAGAAGGCAGG	aaacCCTGCCTTCTCCTCACTCTT
DMD	accgGTCAATGCTCTCCTTTTCAC	aaacGTGAAAAGGAGAGCATTGAC
RUNX1	accgCATGTACTCACCTCTCATGA	aaacTCATGAGAGGTGAGTACATG
GFAP	accgCCAGAATCCAATCTCCCTCA	aaactgagggagattggattctgg
pSnPE-sgRNA	Forward	Reverse

RUNX1	accggcattttcaggaggaagcga	aaactcgcttcctcctgaaaatgc
pSnPE-5'(3')-pRNA	Forward	Reverse
RUNX1	accgtgtctgaagcaatcgcttcctcctgaaaat	aaacattttcaggaggaagcgattgcttcagaca
RUNX1-MS2	accgtgtctgaagcaatcgcttcctcctgaaaatacatgaggatcacccatgt	aaacacatgggtgatcctcatgtattttcaggaggaagcgattgcttcagaca
MS2-RUNX1	accgacatgaggatcacccatgttgtctgaagcaatcgcttcctcctgaaaat	aaactgtctgaagcaatcgcttcctcctgaaaatacatgaggatcacccatgt
RUNX1-PP7	accgtgtctgaagcaatcgcttcctcctgaaaatggagcagacgatatggcgtcgctcc	aaacacatgggtgatcctcatgtattttcaggaggaagcgattgcttcagaca
PP7-RUNX1	accgggagcagacgatatggcgtcgctcctgtctgaagcaatcgcttcctcctgaaaat	aaactgtctgaagcaatcgcttcctcctgaaaatacatgaggatcacccatgt
COM-RUNX1	accgcTGAATGCCTGCGAGCATCtgtctgaagccatcccttcctcctgaaaat	aaacattttcaggaggaagggatggcttcagacaGATGCTCGCAGGCATTCAg
Csy4-RUNX1	accgactgccgtataggcagctaagaaatgtctgaagcaatcgcttcctcctgaaaat	aaacattttcaggaggaagcgattgcttcagacatttcttagctgcctatacggcagt
Boxb-RUNX1	accgggccctgaagaagggcctgtctgaagccatcccttcctcctgaaaat	aaacattttcaggaggaagggatggcttcagacaggcccttcttcagggcc

Supplementary Table 2 | Primers used for cell genomic DNA amplification and targeted deep sequencing.

gene	Forward primer	Reverse primer	Length of amplicon (nt)
JAK2	ctacacgacgctcttccgatctgTGGCGGCATGATTTTGTGCACG	agacgtgtgctcttccgatctccaagcattttagataaggagtacttaactagtat	290
HDAC1	ctacacgacgctcttccgatcttcatgcccagCAAGTGCTGTGAAAC	agacgtgtgctcttccgatctgacactcaccgagcacatcctagcc	290
EED	ctacacgacgctcttccgatctagttggagagagtatgttttctatacttgagatga	agacgtgtgctcttccgatctaactagatacaggattctcttgggtgatagtg	290
BCL11A	ctacacgacgctcttccgatcttattttaatggaattgaaatgtgatatcctgtggtttatgatg	agacgtgtgctcttccgatctccttcagtacttaaaaagtaagggcaatttccagaaa	290
SRD5A3	ctacacgacgctcttccgatcttgcctcgcttgttttccttttgcagAT	agacgtgtgctcttccgatctgcccttctgaggaggtgccg	290
SRD5A1	ctacacgacgctcttccgatctgaaattttacggtttattagccataatcatcttgcaa	agacgtgtgctcttccgatctgatgacttacactgaaaacttgtagctaataactactacg	290
DYRK1A	ctacacgacgctcttccgatctcttgtcacacacaatgaaactttgctgttcact	agacgtgtgctcttccgatctagtagctaacatttttcagtacttaactatatgccagctat	290
DMD	ctacacgacgctcttccgatctAAGCATTAAATCTTAAGACTACAAGACATTACTTGAAGG	agacgtgtgctcttccgatctGACAGAGAAGGGTGTAAAAGCTTCTAGC	290
RUNX1	ctacacgacgctcttccgatcttggttttcgctccgaagGTAAAAGAA	agacgtgtgctcttccgatctatatgcctcagtttgaattcctctcacaaacaag	290
GFAP	ctacacgacgctcttccgatcttactggggaaagcagtgcaggagca	agacgtgtgctcttccgatctaccaccgcttcacagctgtgc	290

Table S3 Sequence of amplicon for deep sequencing analysis

Gene	Sequence
JAK2	gTGGCGGCATGATTTTGTGCACGGATGGATAAAAGTACCTGTGACTCATGAAACACAGGAAGAATGTCTTGGGATGGCAGTGTTAGATATGATGAGAATAGCCAAAGAAAACGATCAAACCCCACTGGCCATCTATAACTCTATCAGgtaattttcttttgcaaatccttacacataagtgtgagtagagattttatataattcgtatatattttctgtgtttacccatgccttttgattttgtaatactagttaagtactccttatctaaaatgcttgg
HDAC1	tcatgcccagCAAGTGCTGTGAAACTTAATAAGCAGCAGACGGACATCGCTGTGAATTGGGCTGGGGGCCTGCACCATGCAAAGAAGTCCGAGGCATCTGGCTTCTGTTACGTCAATGATATCGTCTTGGCCATCCTGGAACTGCTAAAgtatgcctgcctggccttgtctcttggaagagcaccttaggccaggttcccatttccctcttcccctgggcttgcctccctagtttgcttttcctaccgatgtgctggctaggatgtgctcggtgagtgtc
EED	agttggagagagtatgttttctatacttgagatgaaatttactgtatttttattttcattagGTTACCTTGTATGAATGTCATTCACAAGGAGAAATCCGGTTGTTGCAATCTTACGTGGATGCTGATgtatcctttcctgggtttttaatatctttagtcaaaggaatttattttcaataagtgcaccaaaacttttataaattaatcatttccctaaagttattgttctttcttaaccgatttcttcactatcacccaagagaatcctgtatctagtt
BCL11A	tattttaatggaattgaaatgtgatatcctgtggtttatgatgcacgttgtttgtagctgtagtgcttgattttgggtttctttcacagATAAACTTCTGCACTGGAGGGGCCTCTCCTCCCCTCGTTCTGCACATGGAGCTCTAATCCCCACGCCTGGGATGAGTGCAGAATATGCCCCGCAGGGTATTTgtaagttgagccttatttcttctacaaatgtccatgtgtatagagatgagaatttctggaaattgcccttactttttaagtactgaagg

SRD5A3	tgcctcgcttgttttccttttgcagATATTTTTCCCACTTTTATATCATCTCAGTGCTGTGGAATGGCTTCCTGCTTTGGTGC<br>CTTACTCAATCTCTGTTCCTGGGAGCACCTTTTCCAAGCTGGCTTCATGGTTTGCTCAGAATTCTCGGGGCGGCA<br>CAGTTCCAGGgtaaggactccctgggcttatgacaacgctgcatcccgtttctgttgttcctgtctgcaagggagcagtttttggcattttgtctc<br>ctctctcggcacctcctcagaagggc
SRD5A1	gaaattttacggtttattagccataatcatcttgcaattttttcctttagGTTTTGGCTTGTGGTTAACGGGCATGTTGATAAACAT<br>CCATTCAGATCATATCCTAAGGAATCTCAGAAAACCAGGAGATACTGGATACAAAATACCAAGGGgtacgtacaga<br>aagtgaagaatttctgtgaaagttgcttgccatggttcctggctattttagtgttgccagctctaagaagtagtagcgtagtagttattagctacaag<br>ttttcagtgtaagtcatc
DYRK1<br>A	cttgtcacacacaatgaaactttgctgttcactgtcagttataacttacatgaggtgacccatttccattcaagggttttagaagcacatcaaggaca<br>ttctaaggatgattgacttacacaatgatctctgaacatgcctcctgccttctcctcactcttgagtatttgcttaggggagcagaaaaaatactggt<br>agcattactgtaacattttaattctgctttatttaaaaaagacatagctggcatatagttaagtactgaaaaatgttagctact
DMD	AAGCATTAAATCTTAAGACTACAAGACATTACTTGAAGGTCAATGCTCTCCTTTTCACAGGCTACTCAAAAAGAG<br>ATTGAGAAACAGAAGGTGCACCTGAAGAGTATCACAGAGGTAGGAGAGGCCTTGAAAACAGTTTTGGGCAAG<br>AAGGAGACGTTGGTGGAAGATAAACTCAGTCTTCTGAATAGTAACTGGATAGCTGTCACCTCCCGAGCAGAAG<br>AGTGGTTAAATCTTTTGTTGGTAAGAGAAAAGGCTAGAAGCTTTTACACCCTTCTCTGTC
RUNX1	tggttttcgctccgaagGTAAAAGAAATCATTGAGTCCCCCGCCTTCAGAAGAGGGTGCATTTTCAGGAGGAAGCGA<br>TGGCTTCAGACAGCATATTTGAGTCATTTCCTTCGTACCCACAGTGCTTCATGAGAGgtgagtacatgctggtcttgtaat<br>atctacttttgctcagctttgcctgtaatgaaatggcagcttgtttcacctcggtgcagagatgcctcggtgcctgccagttccctgtcttgtttgtgag<br>aggaattcaaactgaggcatat
GFAP	tactggggaaagcagtgcaggagcagcggggcccctgtgtttcattcatggctgggctttgtgactgtgggcagcgagctcacctattctgagcctg<br>tgtccatataaaggaggagttggaagcggagaaggttgatgtccatgagggagattggattctggggtgaagaaagtgagggaaagagcaggc<br>aggtctgggcgcaaagcacaggtgactgcctgccaccagcttgtgaccccccatcaagttactttgacttgcacagctgtgaagcggtggt

Supplementary Sequences S1. Sequence of backbone plasmid used for pegRNA and nicking sgRNA cloning
pDONOR 5.1

acggatcgggagatctaccggtacgcgttgacgtgtcttcggatccggcttactaaaagccagataacagtatgcgtatttgcgcgctgatttttgcggtataagaatatatactgatatgtatac
ccgaagtatgtcaaaaagaggtatgctatgaagcagcgtattacagtgacagttgacagcgacagctatcagttgctcaaggcatatatgatgtcaatatctccggtctggtaagcacaacc
atgcagaatgaagcccgtcgtctgcgtgccgaacgctggaaagcggaaaatcaggaagggatggctgaggtcgcccggtttattgaaatgaacggctcttttgctgacgagaacagggg
ctggtgaaatgcagtttaaggtttacacctataaaagagagagccgttatcgtctgtttgtggatgtacagagtgatattattgacacgcccgggcgacggatggtgatccccctggccagtgc
acgtctgctgtcagataaagtctcccgtgaactttacccggtggtgcatatcggggatgaaagctggcgcatgatgaccaccgatatggccagtgtgccggtctccgttatcggggaagaag
tggctgatctcagccaccgcgaaaatgacatcaaaaacgccattaacctgatgttctggggaatataaatgtcaggctcccttatacacagccagtctgcaggtcgacgaagacccactcg
cggccgcttggggttgcgccttttccaaggcagccctgggtttgcgcagggacgcggctgctctgggcgtggttccgggaaacgcagcggcgccgaccctgggtctcgcacattcttcacgt
ccgttcgcagcgtcacccggatcttcgccgctacccttgtgggccccccggcgacgcttcctgctccgcccctaagtcgggaaggttccttgcggttcgcggcgtgccggacgtgacaaac
ggaagccgcacgtctcactagtaccctcgcagacggacagcgccagggagcaatggcagcgcgccgaccgcgatgggctgtggccaatagcggctgctcagcagggcgcgccgag
agcagcggccgggaaggggcggtgcgggaggcggggtgtggggcggtagtgtgggccctgttcctgcccgcgcggtgttccgcattctgcaagcctccggagcgcacgtcggcagtcg
gctccctcgttgaccgaatcaccgacctctctccccagggggatccgccaccatgaccgagtacaagcccacggtgcgcctcgccacccgcgacgacgtccccagggccgtacgcacc
ctcgccgccgcgttcgccgactaccccgccacgcgccacaccgtcgatccggaccgccacatcgagcgggtcaccgagctgcaagaactcttcctcacgcgcgtcgggctcgacatcg
gcaaggtgtgggtcgcggacgacggcgccgcggtggcggtctggaccacgccggagagcgtcgaagcgggggcggtgttcgccgagatcggcccgcgcatggccgagttgagcgg
ttcccggctggccgcgcagcaacagatggaaggcctcctggcgccgcaccggcccaaggagcccgcgtggttcctggccaccgtcggcgtctcgcccgaccaccagggcaagggtct
gggcagcgccgtcgtgctccccggagtggaggcggccgagcgcgccggggtgcccgccttcctggagacctccgcgccccgcaacctccccttctacgagcggctcggcttcaccgtc
accgccgacgtcgaggtgcccgaaggaccgcgcacctggtgcatgacccgcaagcccggtgccgctagcggtgctactaacttcagcctgctgaagcaggctggagacgtggagga
gaaccctggacctggtagtggaacgcgtatggtgtctaagggcgaagagctgattaaggagaacatgcacatgaagctgtacatggagggcaccgtggacaaccatcacttcaagtgc
acatccgagggcgaaggcaagccctacgagggcacccagaccatgagaatcaaggtggtcgagggcggccctctccccttcgccttcgacatcctggctactagcttcctctacggcag
caagaccttcatcaaccacacccagggcatccccgacttcttcaagcagtccttccctgagggcttcacatgggagagagtcaccacatacgaagatgggggcgtgctgaccgctaccc
aggacaccagcctccaggacggctgcctcatctacaacgtcaagatcagaggggtgaacttcacatccaacggccctgtgatgcagaagaaaacactcggctgggaggccttcaccg
agacgctgtaccccgctgacggcggcctggaaggcagaaacgacatggccctgaagctcgtgggcgggagccatctgatcgcaaacgccaagaccacatatagatccaagaaacc
cgctaagaacctcaagatgcctggcatctactatgtggactacagactggaaagaatcaaggaggccaacaacgaaacctacgtcgagcagcacgaggtggcagtggccagatactg
cgacctccctagcaaactggggcacaagcttaatccaaagaaaaagcggaaagtgtgagtcgacctcgagggggggcccggtacctttaagaccaatgacttacaaggcagctgtag
atcttagccacatgtcatcttattattcagtatttataacttgcaaagaaatgaatatcagagagtgagaggaacttgtttattgcagcttataatggttacaaataaagcaatagcatcacaaattt
cacaaataaagcatttttttcactgcattctagttgtggtttgtccaaactcatcaatgtatcttatcatgtctggaattcacatgtgagcaaaaggccagcaaaaggccaggaaccgtaaaaag
gccgcgttgctggcgtttttccataggctccgcccccctgacgagcatcacaaaaatcgacgctcaagtcagaggtggcgaaacccgacaggactataaagataccaggcgtttcccct
ggaagctccctcgtgcgctctcctgttccgaccctgccgcttaccggatacctgtccgcctttctcccttcgggaagcgtggcgctttctcatagctcacgctgtaggtatctcagttcggtgtaggt
cgttcgctccaagctgggctgtgtgcacgaaccccccgttcagcccgaccgctgcgccttatccggtaactatcgtcttgagtccaacccggtaagacacgacttatcgccactggcagcag
ccactggtaacaggattagcagagcgaggtatgtaggcggtgctacagagttcttgaagtggtggcctaactacggctacactagaagaacagtatttggtatctgcgctctgctgaagcca
gttaccttcggaaaaagagttggtagctcttgatccggcaaacaaaccaccgctggtagcggtttttttgtttgcaagcagcagattacgcgcagaaaaaaaggatctcaagaagatcctttg
atcttttctacggggtctgacgctcagtggaacgaaaactcacgttaagggattttggtcatgagattatcaaaaaggatcttcacctagatccttttaaattaaaaatgaagttttaaatcaatct
aaagtatatatgagtaaacttggtctgacagttaccaatgcttaatcagtgaggcacctatctcagcgatctgtctatttcgttcatccatagttgcctgactccccgtcgtgtagataactacgata
cgggagggcttaccatctggccccagtgctgcaatgataccgcgagaaccacgctcaccggctccagatttatcagcaataaaccagccagccggaagggccgagcgcagaagtggt
cctgcaactttatccgcctccatccagtctattaattgttgccgggaagctagagtaagtagttcgccagttaatagtttgcgcaacgttgttgccattgctacaggcatcgtggtgtcacgctcgtc
gtttggtatggcttcattcagctccggttcccaacgatcaaggcgagttacatgatcccccatgttgtgcaaaaaagcggttagctccttcggtcctccgatcgttgtcagaagtaagttggccgc
agtgttatcactcatggttatggcagcactgcataattctcttactgtcatgccatccgtaagatgcttttctgtgactggtgagtactcaaccaagtcattctgagaatagtgtatgcggcgaccga

gttgctcttgcccggcgtcaatacgggataataccgcgccacatagcagaactttaaaagtgctcatcattggaaaacgttcttcggggcgaaaactctcaaggatcttaccgctgttgagat
ccagttcgatgtaacccactcgtgcacccaactgatcttcagcatcttttactttcaccagcgtttctgggtgagcaaaaacaggaaggcaaaatgccgcaaaaaagggaataagggcga
cacggaaatgttgaatactcatactcttcctttttcaatattattgaagcatttatcagggttattgtctcatgagcggatacatatttgaatgtatttagaaaaataaacaaataggggttccgcgca
catttccccgaaaagtgccacctgacgtc

pLH-AAAA
ttggggttgcgccttttccaaggcagccctgggtttgcgcagggacgcggctgctctgggcgtggttccgggaaacgcagcggcgccgaccctgggtctcgcacattcttcacgtccgttcgc
agcgtcacccggatcttcgccgctacccttgtgggccccccggcgacgcttcctgctccgcccctaagtcgggaaggttccttgcggttcgcggcgtgccggacgtgacaaacggaagcc
gcacgtctcactagtaccctcgcagacggacagcgccagggagcaatggcagcgcgccgaccgcgatgggctgtggccaatagcggctgctcagcagggcgcgccgagagcagcg
gccgggaaggggcggtgcgggaggcggggtgtggggcggtagtgtgggccctgttcctgcccgcgcggtgttccgcattctgcaagcctccggagcgcacgtcggcagtcggctccctc
gttgaccgaatcaccgacctctctccccagggggatccaccggatcaagcttgccagctggggcgccctctggtaaggttgggaagccctgcagatctgatcccgggggcaatgagat
atgaaaaagcctgaactcaccgcgacgtctgtcgagaagtttctgatcgaaaagttcgacagcgtctccgacctgatgcagctctcggagggcgaagaatctcgtgctttcagcttcgatgt
aggagggcgtggatatgtcctgcgggtaaatagctgcgccgatggtttctacaaagatcgttatgtttatcggcactttgcatcggccgcgctcccgattccggaagtgcttgacattggggaa
tttagcgagagcctgacctattgcatctcccgccgtgcacagggtgtcacgttgcaagatctgcctgaaaccgaactgcccgctgttctgcagccggtcgcggaggccatggatgcgatcgc
tgcggccgatcttagccagacgagcgggttcggcccattcggaccgcaaggaatcggtcaatacactacatggcgtgatttcatatgcgcgattgctgatccccatgtgtatcactggcaaa
ctgtgatggacgacaccgtcagtgcgtccgtcgcgcaggctctcgatgagctgatgctttgggccgaggactgccccgaagtccggcacctcgtgcacgcggatttcggctccaacaatgt
cctgacggacaatggccgcataacagcggtcattgactggagcgaggcgatgttcggggattcccaatacgaggtcgccaacatcttcttctggaggccgtggttggcttgtatggagcag
cagacgcgctacttcgagcggaggcatccggagcttgcaggatcgccgcggctccgggcgtatatgctccgcattggtcttgaccaactctatcagagcttggttgacggcaatttcgatgat
gcagcttgggcgcagggtcgatgcgacgcaatcgtccgatccggagccgggactgtcgggcgtacacaaatcgcccgcagaagcgcggccgtctggaccgatggctgtgtagaagta
ctcgccgatagtggaaaccgacgccccagcactcgtccgagggcaaaggaatagagtagatgccgaccgggatctatcgataccgtcgacctcgagggggggcccggtacctttaag
accaatgacttacaaggcagctgtagatcttagccactttttaaaagaaaaggggggactggaagggctaattcactcccaacgaagaaaagatctgctttttgcttgtactgggtctctctgg
ttagaccagatctgagcctgggagctctctggctaactagggaacccactgcttaagcctcaataaagcttgccttgagtgcttcaagtagtgtgtgcccgtctgttgtgtgactctggtaactag
agatccctcagacccttttagtcagtgtggaaaatctctagcagtagtagttcatgtcatcttattattcagtatttataacttgcaaagaaatgaatatcagagagtgagaggaacttgtttattgc
agcttataatggttacaaataaagcaatagcatcacaaatttcacaaataaagcatttttttcactgcattctagttgtggtttgtccaaactcatcaatgtatcttatcatgtctggctctagctatcc
cgcccctaactccgcccatcccgcccctaactccgcccagttccgcccattctccgccccatggctgactaattttttttatttatgcagaggccgaggccgcctcggcctctgagctattccag
aagtagtgaggaggcttttttggaggcctagggacgtacccaattcgccctatagtgagtcgtattacgcgcgctcactggccgtcgttttacaacgtcgtgactgggaaaaccctggcgttac
ccaacttaatcgccttgcagcacatccccctttcgccagctggcgtaatagcgaagaggcccgcaccgatcgcccttcccaacagttgcgcagcctgaatggcgaatggacgcgccctg
tagcggcgcattaagcgcggcgggtgtggtggttacgcgcagcgtgaccgctacacttgccagcgccctagcgcccgctcctttcgctttcttcccttcctttctcgccacgttcgccggctttcc
ccgtcaagctctaaatcgggggctccctttagggttccgatttagtgctttacggcacctcgaccccaaaaaacttgattagggtgatggttcacgtagtgggccatcgccctgatagacggttt
ttcgccctttgacgttggagtccacgttctttaatagtggactcttgttccaaactggaacaacactcaaccctatctcggtctattcttttgatttataagggattttgccgatttcggcctattggttaa
aaaatgagctgatttaacaaaaatttaacgcgaattttaacaaaatattaacgcttacaatttaggtggcacttttcggggaaatgtgcgcggaacccctatttgtttatttttctaaatacattcaa
atatgtatccgctcatgagacaataaccctgataaatgcttcaataatattgaaaaaggaagagtatgagtattcaacatttccgtgtcgcccttattcccttttttgcggcattttgccttcctgttttt
gctcacccagaaacgctggtgaaagtaaaagatgctgaagatcagttgggtgcacgagtgggttacatcgaactggatctcaacagcggtaagatccttgagagttttcgccccgaagaa
cgttttccaatgatgagcacttttaaagttctgctatgtggcgcggtattatcccgtattgacgccgggcaagagcaactcggtcgccgcatacactattctcagaatgacttggttgagtactca
ccagtcacagaaaagcatcttacggatggcatgacagtaagagaattatgcagtgctgccataaccatgagtgataacactgcggccaacttacttctgacaacgatcggaggaccgaa
ggagctaaccgcttttttgcacaacatgggggatcatgtaactcgccttgatcgttgggaaccggagctgaatgaagccataccaaacgacgagcgtgacaccacgatgcctgtagcaat
ggcaacaacgttgcgcaaactattaactggcgaactacttactctagcttcccggcaacaattaatagactggatggaggcggataaagttgcaggaccacttctgcgctcggcccttccgg
ctggctggtttattgctgataaatctggagccggtgagcgtgggtctcgcggtatcattgcagcactggggccagatggtaagccctcccgtatcgtagttatctacacgacggggagtcagg
caactatggatgaacgaaatagacagatcgctgagataggtgcctcactgattaagcattggtaactgtcagaccaagtttactcatatatactttagattgatttaaaacttcatttttaatttaaa
aggatctaggtgaagatcctttttgataatctcatgaccaaaatcccttaacgtgagttttcgttccactgagcgtcagaccccgtagaaaagatcaaaggatcttcttgagatcctttttttctgcg
cgtaatctgctgcttgcaaacaaaaaaaccaccgctaccagcggtggtttgtttgccggatcaagagctaccaactctttttccgaaggtaactggcttcagcagagcgcagataccaaata
ctgttcttctagtgtagccgtagttaggccaccacttcaagaactctgtagcaccgcctacatacctcgctctgctaatcctgttaccagtggctgctgccagtggcgataagtcgtgtcttaccgg
gttggactcaagacgatagttaccggataaggcgcagcggtcgggctgaacggggggttcgtgcacacagcccagcttggagcgaacgacctacaccgaactgagatacctacagcg
tgagctatgagaaagcgccacgcttcccgaagggagaaaggcggacaggtatccggtaagcggcagggtcggaacaggagagcgcacgagggagcttccagggggaaacgcct
ggtatctttatagtcctgtcgggtttcgccacctctgacttgagcgtcgatttttgtgatgctcgtcaggggggcggagcctatggaaaaacgccagcaacgcggcctttttacggttcctggcctt
ttgctggccttttgctcacatgttctttcctgcgttatcccctgattctgtggataaccgtattaccgcctttgagtgagctgataccgctcgccgcagccgaacgaccgagcgcagcgagtcagtg
agcgaggaagcggaagagcgcccaatacgcaaaccgcctctccccgcgcgttggccgattcattaatgcagctggcacgacaggtttcccgactggaaagcgggcagtgagcgcaa
cgcaattaatgtgagttagctcactcattaggcaccccaggctttacactttatgcttccggctcgtatgttgtgtggaattgtgagcggataacaatttcacacaggaaacagctatgaccatga
ttacgccaagcgcgcaattaaccctcactaaagggaacaaaagctggagctgcaagcttaatgtagtcttatgcaatactcttgtagtcttgcaacatggtaacgatgagttagcaacatgcc
ttacaaggagagaaaaagcaccgtgcatgccgattggtggaagtaaggtggtacgatcgtgccttattaggaaggcaacagacgggtctgacatggattggacgaaccactgaattgcc
gcattgcagagatattgtatttaagtgcctagctcgatacataaacgggtctctctggttagaccagatctgagcctgggagctctctggctaactagggaacccactgcttaagcctcaataa
agcttgccttgagtgcttcaagtagtgtgtgcccgtctgttgtgtgactctggtaactagagatccctcagacccttttagtcagtgtggaaaatctctagcagtggcgcccgaacagggacttga
aagcgaaagggaaaccagaggagctctctcgacgcaggactcggcttgctgaagcgcgcacggcaagaggcgaggggcggcgactggtgagtacgccaaaaattttgactagcgg
aggctagaaggagagagatgggtgcgagagcgtcagtattaagcgggggagaattagatcgcgatgggaaaaaattcggttaaggccagggggaaagaaaaaatataaattaaaa
catatagtatgggcaagcagggagctagaacgattcgcagttaatcctggcctgttagaaacatcagaaggctgtagacaaatactgggacagctacaaccatcccttcagacaggatc
agaagaacttagatcattatataatacagtagcaaccctctattgtgtgcatcaaaggatagagataaaagacaccaaggaagctttagacaagatagaggaagagcaaaacaaaagt
aagaccaccgcacagcaagcggccgctgatcttcagacctggaggaggagatatgagggacaattggagaagtgaattatataaatataaagtagtaaaaattgaaccattaggagta
gcacccaccaaggcaaagagaagagtggtgcagagagaaaaaagagcagtgggaataggagctttgttccttgggttcttgggagcagcaggaagcactatgggcgcagcgtcaat
gacgctgacggtacaggccagacaattattgtctggtatagtgcagcagcagaacaatttgctgagggctattgaggcgcaacagcatctgttgcaactcacagtctggggcatcaagca
gctccaggcaagaatcctggctgtggaaagatacctaaaggatcaacagctcctggggatttggggttgctctggaaaactcatttgcaccactgctgtgccttggaatgctagttggagtaa
taaatctctggaacagatttggaatcacacgacctggatggagtgggacagagaaattaacaattacacaagcttaatacactccttaattgaagaatcgcaaaaccagcaagaaaaga
atgaacaagaattattggaattagataaatgggcaagtttgtggaattggtttaacataacaaattggctgtggtatataaaattattcataatgatagtaggaggcttggtaggtttaagaatag
tttttgctgtactttctatagtgaatagagttaggcagggatattcaccattatcgtttcagacccacctcccaaccccgaggggacccgacaggcccgaaggaatagaagaagaaggtgga
gagagagacagagacagatccattcgattagtgaacggatctcgacggtatcgatcacgagactagcctcgagcggccgccccccttcaccgagggcctatttcccatgattccttcatattt
gcatatacgatacaaggctgttagagagataattggaattaatttgactgtaaacacaaagatattagtacaaaatacgtgacgtagaaagtaataatttcttgggtagtttgcagttttaaaatt
atgttttaaaatggactatcatatgcttaccgtaacttgaaagtatttcgatttcttggctttatatatcttgtggaaaggacgaaacaccggtgtcttcgaggcttactaaaagccagataacagta
tgcgtatttgcgcgctgatttttgcggtataagaatatatactgatatgtatacccgaagtatgtcaaaaagaggtgtgctatgaagcagcgtattacagtgacagttgacagcgacagctatca
gttgctcaaggcatatatgatgtcaatatctccggtctggtaagcacaaccatgcagaatgaagcccgtcgtctgcgtgccgaacgctggaaagcggaaaatcaggaagggatggctga
ggtcgcccggtttattgaaatgaacggctcttttgctgacgagaacagggactggtgaaatgcagtttaaggtttacacctataaaagagagagccgttatcgtctgtttgtggatgtacagagt
gatattattgacacgcccgggcgacggatggtgatccccctggccagtgcacgtctgctgtcagataaagtctcccgtgaactttacccggtggtgcatatcggggatgaaagctggcgcat

gatgaccaccgatatggccagtgtgccggtctccgttatcggggaagaagtggctgatctcagccaccgcgaaaatgacatcaaaaacgccattaacctgatgttctggggaatataaac
cctgcaggacgaagacccttttttgaattctcgacctcgagacaaatggcagtattcatccacaattttaaaagaaaaggggggattggggggtacagtgcaggggaaagaatagtaga
cataatagcaacagacatacaaactaaagaattacaaaaacaaattacaaaaattcaaaattttcgggtttattacagggacagcagagatccactttggccgcggctcgagggggg

pLH-sgRNA3

ttggggttgcgccttttccaaggcagccctgggtttgcgcagggacgcggctgctctgggcgtggttccgggaaacgcagcggcgccgaccctgggtctcgcacattcttcacgtccgttcgc
agcgtcacccggatcttcgccgctacccttgtgggccccccggcgacgcttcctgctccgcccctaagtcgggaaggttccttgcggttcgcggcgtgccggacgtgacaaacggaagcc
gcacgtctcactagtaccctcgcagacggacagcgccagggagcaatggcagcgcgccgaccgcgatgggctgtggccaatagcggctgctcagcagggcgcgccgagagcagcg
gccgggaaggggcggtgcgggaggcggggtgtggggcggtagtgtgggccctgttcctgcccgcgcggtgttccgcattctgcaagcctccggagcgcacgtcggcagtcggctccctc
gttgaccgaatcaccgacctctctccccagggggatccaccggatcaagcttgccagctggggcgccctctggtaaggttgggaagccctgcagatctgatcccgggggcaatgagat
atgaaaaagcctgaactcaccgcgacgtctgtcgagaagtttctgatcgaaaagttcgacagcgtctccgacctgatgcagctctcggagggcgaagaatctcgtgctttcagcttcgatgt
aggagggcgtggatatgtcctgcgggtaaatagctgcgccgatggtttctacaaagatcgttatgtttatcggcactttgcatcggccgcgctcccgattccggaagtgcttgacattggggaa
tttagcgagagcctgacctattgcatctcccgccgtgcacagggtgtcacgttgcaagatctgcctgaaaccgaactgcccgctgttctgcagccggtcgcggaggccatggatgcgatcgc
tgcggccgatcttagccagacgagcgggttcggcccattcggaccgcaaggaatcggtcaatacactacatggcgtgatttcatatgcgcgattgctgatccccatgtgtatcactggcaaa
ctgtgatggacgacaccgtcagtgcgtccgtcgcgcaggctctcgatgagctgatgctttgggccgaggactgccccgaagtccggcacctcgtgcacgcggatttcggctccaacaatgt
cctgacggacaatggccgcataacagcggtcattgactggagcgaggcgatgttcggggattcccaatacgaggtcgccaacatcttcttctggaggccgtggttggcttgtatggagcag
cagacgcgctacttcgagcggaggcatccggagcttgcaggatcgccgcggctccgggcgtatatgctccgcattggtcttgaccaactctatcagagcttggttgacggcaatttcgatgat
gcagcttgggcgcagggtcgatgcgacgcaatcgtccgatccggagccgggactgtcgggcgtacacaaatcgcccgcagaagcgcggccgtctggaccgatggctgtgtagaagta
ctcgccgatagtggaaaccgacgccccagcactcgtccgagggcaaaggaatagagtagatgccgaccgggatctatcgataccgtcgacctcgagggggggcccggtacctttaag
accaatgacttacaaggcagctgtagatcttagccactttttaaaagaaaaggggggactggaagggctaattcactcccaacgaagaaaagatctgctttttgcttgtactgggtctctctgg
ttagaccagatctgagcctgggagctctctggctaactagggaacccactgcttaagcctcaataaagcttgccttgagtgcttcaagtagtgtgtgcccgtctgttgtgtgactctggtaactag
agatccctcagacccttttagtcagtgtggaaaatctctagcagtagtagttcatgtcatcttattattcagtatttataacttgcaaagaaatgaatatcagagagtgagaggaacttgtttattgc
agcttataatggttacaaataaagcaatagcatcacaaatttcacaaataaagcatttttttcactgcattctagttgtggtttgtccaaactcatcaatgtatcttatcatgtctggctctagctatcc
cgcccctaactccgcccatcccgcccctaactccgcccagttccgcccattctccgccccatggctgactaattttttttatttatgcagaggccgaggccgcctcggcctctgagctattccag
aagtagtgaggaggcttttttggaggcctagggacgtacccaattcgccctatagtgagtcgtattacgcgcgctcactggccgtcgttttacaacgtcgtgactgggaaaaccctggcgttac
ccaacttaatcgccttgcagcacatccccctttcgccagctggcgtaatagcgaagaggcccgcaccgatcgcccttcccaacagttgcgcagcctgaatggcgaatggacgcgccctg
tagcggcgcattaagcgcggcgggtgtggtggttacgcgcagcgtgaccgctacacttgccagcgccctagcgcccgctcctttcgctttcttcccttcctttctcgccacgttcgccggctttcc
ccgtcaagctctaaatcgggggctccctttagggttccgatttagtgctttacggcacctcgaccccaaaaaacttgattagggtgatggttcacgtagtgggccatcgccctgatagacggttt
ttcgccctttgacgttggagtccacgttctttaatagtggactcttgttccaaactggaacaacactcaaccctatctcggtctattcttttgatttataagggattttgccgatttcggcctattggttaa
aaaatgagctgatttaacaaaaatttaacgcgaattttaacaaaatattaacgcttacaatttaggtggcacttttcggggaaatgtgcgcggaacccctatttgtttatttttctaaatacattcaa
atatgtatccgctcatgagacaataaccctgataaatgcttcaataatattgaaaaaggaagagtatgagtattcaacatttccgtgtcgcccttattcccttttttgcggcattttgccttcctgtttt
gctcacccagaaacgctggtgaaagtaaaagatgctgaagatcagttgggtgcacgagtgggttacatcgaactggatctcaacagcggtaagatccttgagagttttcgccccgaagaa
cgttttccaatgatgagcacttttaaagttctgctatgtggcgcggtattatcccgtattgacgccgggcaagagcaactcggtcgccgcatacactattctcagaatgacttggttgagtactca
ccagtcacagaaaagcatcttacggatggcatgacagtaagagaattatgcagtgctgccataaccatgagtgataacactgcggccaacttacttctgacaacgatcggaggaccgaa
ggagctaaccgcttttttgcacaacatgggggatcatgtaactcgccttgatcgttgggaaccggagctgaatgaagccataccaaacgacgagcgtgacaccacgatgcctgtagcaat
ggcaacaacgttgcgcaaactattaactggcgaactacttactctagcttcccggcaacaattaatagactggatggaggcggataaagttgcaggaccacttctgcgctcggcccttccgg
ctggctggtttattgctgataaatctggagccggtgagcgtgggtctcgcggtatcattgcagcactggggccagatggtaagccctcccgtatcgtagttatctacacgacggggagtcagg
caactatggatgaacgaaatagacagatcgctgagataggtgcctcactgattaagcattggtaactgtcagaccaagtttactcatatatactttagattgatttaaaacttcatttttaatttaaa
aggatctaggtgaagatcctttttgataatctcatgaccaaaatcccttaacgtgagttttcgttccactgagcgtcagaccccgtagaaaagatcaaaggatcttcttgagatcctttttttctgcg
cgtaatctgctgcttgcaaacaaaaaaaccaccgctaccagcggtggtttgtttgccggatcaagagctaccaactctttttccgaaggtaactggcttcagcagagcgcagataccaaata
ctgttcttctagtgtagccgtagttaggccaccacttcaagaactctgtagcaccgcctacatacctcgctctgctaatcctgttaccagtggctgctgccagtggcgataagtcgtgtcttaccgg
gttggactcaagacgatagttaccggataaggcgcagcggtcgggctgaacggggggttcgtgcacacagcccagcttggagcgaacgacctacaccgaactgagatacctacagcg
tgagctatgagaaagcgccacgcttcccgaagggagaaaggcggacaggtatccggtaagcggcagggtcggaacaggagagcgcacgagggagcttccagggggaaacgcct
ggtatctttatagtcctgtcgggtttcgccacctctgacttgagcgtcgatttttgtgatgctcgtcaggggggcggagcctatggaaaaacgccagcaacgcggcctttttacggttcctggcctt
ttgctggccttttgctcacatgttctttcctgcgttatcccctgattctgtggataaccgtattaccgcctttgagtgagctgataccgctcgccgcagccgaacgaccgagcgcagcgagtcagtg
agcgaggaagcggaagagcgcccaatacgcaaaccgcctctccccgcgcgttggccgattcattaatgcagctggcacgacaggtttcccgactggaaagcgggcagtgagcgcaa
cgcaattaatgtgagttagctcactcattaggcaccccaggctttacactttatgcttccggctcgtatgttgtgtggaattgtgagcggataacaatttcacacaggaaacagctatgaccatga
ttacgccaagcgcgcaattaaccctcactaaagggaacaaaagctggagctgcaagcttaatgtagtcttatgcaatactcttgtagtcttgcaacatggtaacgatgagttagcaacatgcc
ttacaaggagagaaaaagcaccgtgcatgccgattggtggaagtaaggtggtacgatcgtgccttattaggaaggcaacagacgggtctgacatggattggacgaaccactgaattgcc
gcattgcagagatattgtatttaagtgcctagctcgatacataaacgggtctctctggttagaccagatctgagcctgggagctctctggctaactagggaacccactgcttaagcctcaataa
agcttgccttgagtgcttcaagtagtgtgtgcccgtctgttgtgtgactctggtaactagagatccctcagacccttttagtcagtgtggaaaatctctagcagtggcgcccgaacagggacttga
aagcgaaagggaaaccagaggagctctctcgacgcaggactcggcttgctgaagcgcgcacggcaagaggcgaggggcggcgactggtgagtacgccaaaaattttgactagcgg
aggctagaaggagagagatgggtgcgagagcgtcagtattaagcgggggagaattagatcgcgatgggaaaaaattcggttaaggccagggggaaagaaaaaatataaattaaaa
catatagtatgggcaagcagggagctagaacgattcgcagttaatcctggcctgttagaaacatcagaaggctgtagacaaatactgggacagctacaaccatcccttcagacaggatc
agaagaacttagatcattatataatacagtagcaaccctctattgtgtgcatcaaaggatagagataaaagacaccaaggaagctttagacaagatagaggaagagcaaaacaaaagt
aagaccaccgcacagcaagcggccgctgatcttcagacctggaggaggagatatgagggacaattggagaagtgaattatataaatataaagtagtaaaaattgaaccattaggagta
gcacccaccaaggcaaagagaagagtggtgcagagagaaaaaagagcagtgggaataggagctttgttccttgggttcttgggagcagcaggaagcactatgggcgcagcgtcaat
gacgctgacggtacaggccagacaattattgtctggtatagtgcagcagcagaacaatttgctgagggctattgaggcgcaacagcatctgttgcaactcacagtctggggcatcaagca
gctccaggcaagaatcctggctgtggaaagatacctaaaggatcaacagctcctggggatttggggttgctctggaaaactcatttgcaccactgctgtgccttggaatgctagttggagtaa
taaatctctggaacagatttggaatcacacgacctggatggagtgggacagagaaattaacaattacacaagcttaatacactccttaattgaagaatcgcaaaaccagcaagaaaaga
atgaacaagaattattggaattagataaatgggcaagtttgtggaattggtttaacataacaaattggctgtggtatataaaattattcataatgatagtaggaggcttggtaggtttaagaatag
tttttgctgtactttctatagtgaatagagttaggcagggatattcaccattatcgtttcagacccacctcccaaccccgaggggacccgacaggcccgaaggaatagaagaagaaggtgga
gagagagacagagacagatccattcgattagtgaacggatctcgacggtatcgatcacgagactagcctcgagcggccgccccccttcaccgagggcctatttcccatgattccttcatattt
gcatatacgatacaaggctgttagagagataattggaattaatttgactgtaaacacaaagatattagtacaaaatacgtgacgtagaaagtaataatttcttgggtagtttgcagttttaaaatt
atgttttaaaatggactatcatatgcttaccgtaacttgaaagtatttcgatttcttggctttatatatcttgtggaaaggacgaaacaccgggtgtcttcgaggcttactaaaagccagataacagta
tgcgtatttgcgcgctgatttttgcggtataagaatatatactgatatgtatacccgaagtatgtcaaaaagaggtgtgctatgaagcagcgtattacagtgacagttgacagcgacagctatca
gttgctcaaggcatatatgatgtcaatatctccggtctggtaagcacaaccatgcagaatgaagcccgtcgtctgcgtgccgaacgctggaaagcggaaaatcaggaagggatggctga
ggtcgcccggtttattgaaatgaacggctcttttgctgacgagaacagggactggtgaaatgcagtttaaggtttacacctataaaagagagagccgttatcgtctgtttgtggatgtacagagt
gatattattgacacgcccgggcgacggatggtgatcccctggccagtgcacgtctgctgtcagataaagtctcccgtgaactttacccggtggtgcatatcggggatgaaagctggcgcat
gatgaccaccgatatggccagtgtgccggtctccgttatcggggaagaagtggctgatctcagccaccgcgaaaatgacatcaaaaacgccattaacctgatgttctggggaatataaac

cctgcaggacgaagacccgttttagagctagaaatagcaagttaaaataaggctagtccgttatcaacttgaaaaagtggcaccgagtcggtgctttttttgaattctcgacctcgagacaaa
tggcagtattcatccacaattttaaaagaaaaggggggattggggggtacagtgcaggggaaagaatagtagacataatagcaacagacatacaaactaaagaattacaaaaacaaa
ttacaaaaattcaaaattttcgggtttattacagggacagcagagatccactttggccgcggctcgaggggg

Yellow is U6 promoter
Cyan is ccdb
Red sgRNA backbone

Supplementary Sequences S2. Sequences of prime editors (MCP, tdMCP, PCP, COM, N22p, Csy4H29A and PYL1-P2A-ABI)

MCP
Atggcttctaactttactcagttcgttctcgtcgacaatggcggaactggcgacgtgactgtcgccccaagcaacttcgctaacgggatcgctgaatggatcagctctaactcgcgttcacagg
cttacaaagtaacctgtagcgttcgtcagagctctgcgcagaatcgcaaatacaccatcaaagtcgaggtgcctaaaggcgcctggcgttcgtacttaaatatggaactaaccattccaattt
tcgccacgaattccgactgcgagcttattgttaaggcaatgcaaggtctcctaaaagatggaaacccgattccctcagcaatcgcagcaaactccggcatctac

tdMCP
gcttctaactttactcagttcgttctcgtcgacaatggcggaactggcgacgtgactgtcgccccaagcaacttcgctaacgggatcgctgaatggatcagctctaactcgcgttcacaggctt
acaaagtaacctgtagcgttcgtcagagctctgcgcagaatcgcaaatacaccatcaaagtcgaggtgcctaaaggcgcctggcgttcgtacttaaatatggaactaaccattccaattttc
gccacgaattccgactgcgagcttattgttaaggcaatgcaaggtctcctaaaagatggaaacccgattccctcagcaatcgcagcaaactccggcatctacgccatggccagcaacttc
acccagttcgtgctggtggacaacggcggcaccggcgacgtgaccgtggcccccagcaacttcgccaacggcatcgccgagtggatcagcagcaacagcagaagccaggcctaca
aggtgacctgcagcgtgagacagagcagcgcccagaacagaaagtacaccatcaaggtggaggtgcccaagggcgcctggagaagctacctgaacatggagctgaccatccccat
cttcgccaccaacagcgactgcgagctgatcgtgaaggccatgcagggcctgctgaaggacggcaaccccatccccagcgccatcgccgccaacagcggcatctacgcc

PCP
Atgggttccaaaaccatcgttctttcggtcggcgaggctactcgcactctgactgagatccagtccaccgcagaccgtcagatcttcgaagagaaggtcgggcctctggtgggtcggctgcg
cctcacggcttcgctccgtcaaaacggagccaagaccgcgtatcgcgtcaacctaaaactggatcaggcggacgtcgttgattccggacttccgaaagtgcgctacactcaggtatggtc
gcacgacgtgacaatcgttgcgaatagcaccgaggcctcgcgcaaatcgttgtacgatttgaccaagtccctcgtcgcgacctcgcaggtcgaagatcttgtcgtcaaccttgtgccgctgg
gccgt

tdPCP
tccaaaactatcgttctgagcgtaggtgaagcgaccagaacgttaactgaaattcaatctacagcagacaggcaaatatttgaagagaaagtcggtccattagtagggaggttaagattga
cagctagcttaagacaaaatggagctaaaactgcatacagggtcaatttaaaattggatcaagcagatgtcgttgatagcggtctacctaaggttagatacacgcaagtgtggtcacacga
tgtcactattgtcgcgaattctaccgaagctagtagaaaatcattgtatgatttaacaaaatccttggtggctacctctcaggtggaagatcttgttgtgaatttagttccattaggtagggcggacc
cgctagcctccaaaaccatcgttctttcggtcggcgaggctactcgcactctgactgagatccagtccaccgcagaccgtcagatcttcgaagagaaggtcgggcctctggtgggtcggctg
cgcctcacggcttcgctccgtcaaaacggagccaagaccgcgtatcgcgtcaacctaaaactggatcaggcggacgtcgttgattccggacttccgaaagtgcgctacactcaggtatgg
tcgcacgacgtgacaatcgttgcgaatagcaccgaggcctcgcgcaaatcgttgtacgatttgaccaagtccctcgtcgcgacctcgcaggtcgaagatcttgtcgtcaaccttgtgccgctg
ggccgt

COM
ggtagtatgaaatcaattcgctgtaaaaactgcaacaaactgttatttaaggcggatagttttgatcacattgaaatcaggtgtccgcgttgcaaacgtcacatcataatgctgaatgcctgcga
gcatcccacggagaaacattgtgggaaaagagaaaaaatcacgcattctgacgaaaccgtgcgttatggctccactagtgggcacaagcttaatggcggtggcggagggatggatgct
aagtcactaactgcctggtcc

N22p
ggtaatgctcggacccggcgaagagagaggcgggctgagaagcaggcacagtggaaggctgcaaac

Csy4H29A
Atggaccactacctcgacattcgcttgcgaccggacccggaatttcccccggcgcaactcatgagcgtgctcttcggcaagctcgcccaggccctggtggcacagggcggggacaggat
cggcgtgagcttccccgacctcgacgaaagccgctcccggctgggcgagcgcctgcgcattcatgcctcggcggacgaccttcgtgccctgctcgcccggccctggctggaagggttgc
gggaccatctgcaattcggagaaccggcagtcgtgcctcaccccacaccgtaccgtcaggtcagtcgggttcaggcgaaaagcaatccggaacgcctgcggcggcggctcatgcgcc
ggcacgatctgagtgaggaggaggctcggaaacgcattcccgatacggtcgcgagagccttggacctgcccttcgtcacgctacgcagccagagcaccggacagcacttccgtctcttc
atccgccacgggccgttgcaggtgacggcagaggaaggaggattcacctgttacgggttgagcaaaggaggtttcgttccctggttc

PYL1-P2A-ABI
ggtgggggcgcgccaactcaagacgaattcacccaactctcccaatcaatcgccgagttccacacgtaccaactcggtaacggccgttgctcatctctcctagctcagcgaatccacgcg
ccgccggaaacagtatggtccgtggtgagacgtttcgataggccacagatttacaaacacttcatcaaaagctgtaacgtgagtgaagatttcgagatgcgagtgggatgcacgcgcgac
gtgaacgtgataagtggattaccggcgaatacgtctcgagagagattagatctgttggacgatgatcggagagtgactgggtttagtataaccggtggtgaacataggctgaggaattataa
atcggttacgacggttcatagatttgagaaagaagaagaagaagaaaggatctggaccgttgttttggaatcttatgttgttgatgtaccggaaggtaattcggaggaagatacgagattgttt
gctgatacggttattagattgaatcttcagaaacttgcttcgatcactgaagctatgaacggaggcagtgctactaacttcagcctgctgaagcaggctggagacgtggaggagaaccctgg
acctgggggttccacgcgtgtgcctttgtatggttttacttcgatttgtggaagaagacctgagatggaagctgctgtttcgactataccaagattccttcaatcttcctctggttcgatgttagatggt
cggtttgatcctcaatccgccgctcatttcttcggtgtttacgacggccatggcggttctcaggtagcgaactattgtagagagaggatgcatttggctttggcggaggagatagctaaggaga
aaccgatgctctgcgatggtgatacgtggctggagaagtggaagaaagctcttttcaactcgttcctgagagttgactcggagattgagtcagttgcgccggagacggttgggtcaacgtcg
gtggttgccgttgttttcccgtctcacatcttcgtcgctaactgcggtgactctagagccgttctttgccgcggcaaaactgcacttccattatccgttgaccataaaccggatagagaagatgaa
gctgcgaggattgaagccgcaggagggaaagtgattcagtggaatggagctcgtgttttcggtgttctcgccatgtcgagatccattggcgatagatacttgaaaccatccatcattcctgatc
cggaagtgacggctgtgaagagagtaaaagaagatgattgtctgattttggcgagtgacggggtttgggatgtaatgacggatgaagaagcgtgtgagatggcaaggaagcggattctct
tgtggcacaagaaaaacgcggtggctggggatgcatcgttgctcgcggatgagcggagaaaggaagggaaagatcctgcggcgatgtccgcggctgagtatttgtcaaagctggcgat
acagagaggaagcaaagacaacataagtgtggtggtggttgatttgaag
